# Systematic study and 30-year projections of global and multi-regional burden of multiple sclerosis, 1990–2021

**DOI:** 10.1097/MD.0000000000045089

**Published:** 2025-10-31

**Authors:** Jie Liu, Huasheng Fan, Han Wang, Yinfu Xu, Yuchen Guo, Peijun Han, Yuezhen Shen

**Affiliations:** a Department of Neurosurgery, The Second People’s Hospital of Liaocheng, Liaocheng, Shandong, China; b Department of Neurosurgery, The First People’s Hospital of Nanning, Nanning, China; c Department of Neurology, The Second People’s Hospital of Liaocheng, Liaocheng, Shandong, China.

**Keywords:** GBD database, multiple sclerosis, prediction

## Abstract

Multiple sclerosis (MS) is a chronic neurodegenerative disease with significant temporal and regional heterogeneity. While earlier studies described the burden before 2019, recent shifts influenced by socioeconomic development, healthcare access, and environmental exposures remain unclear. Using the Global Burden of Disease database, we analyzed recent MS trends, assessed interactions of gender, age, and sociodemographic index (SDI), and projected future dynamics. Based on the Global Burden of Disease database from 1990 to 2021, this study collected epidemiological data of 2795 patients with MS from 204 countries and regions, covering core indicators such as incidence, prevalence, mortality, and disability-adjusted life years (DALYs). Data underwent standardized processing and WHO age-standardization. Long-term trends were analyzed with Joinpoint regression; regional variation by SDI and Moran *I*; gender and age distributions with chi-square; and inequalities by concentration indices and Lorenz curves. A Bayesian hierarchical model with Markov chain Monte Carlo was applied to forecast trends to 2050. From 1990 to 2021, global MS cases rose markedly (incidence +49.9%, prevalence +87.9%), but age-standardized incidence and prevalence remained stable (−3.5% and −0.4%), indicating population growth as the main driver. High-SDI regions showed rising incidence (Western Europe +27.8%, Latin America +31.6%), while low-SDI regions had sharp increases in case numbers but limited standardized rate changes. Mortality and DALYs decreased globally (−12.8% and −11.0%) but rose in resource-limited areas (mortality +110.9% in Central Latin America, DALYs +315% in West Africa). Women consistently bore a higher burden, with gender gaps most evident in low-income regions (315% higher mortality in West African women). MS prevalence strongly correlated with SDI (*r* = 0.6975, *P* < .001). Projections suggest gradual incidence growth with declining mortality and DALYs by 2050. Inequality analysis showed persistent deviations from equilibrium. Despite improved survival, high-SDI regions face the challenge of managing aging patients, while low-SDI regions suffer from high mortality and limited resources. The disproportionate burden in women, especially in low-income settings, underscores the need for tailored, equity-focused strategies.

## 1. Introduction

Multiple sclerosis (MS) is one of the most common neuroinflammatory diseases in the world.^[1]^ The onset of MS is characterized by younger age, and the average age of diagnosis is between 20 and 40 years old. The time window for the progression of MS is wide, but mostly within 20 years.^[2]^ The factors leading to MS are complex. Genetic and environmental factors have been confirmed to be related to the pathogenesis of MS, but the specific pathogenesis of MS remains to be studied.^[3]^ At present, the treatment of MS only focuses on relieving neurological symptoms and reducing recurrence, and there is still no detailed research report on the long-term efficacy of such drugs.^[4]^ It is worth noting that the use of disease-modifying therapies (DMTs) for MS, especially for the relapsing-remitting type of MS, has produced favorable clinical outcomes, helping to reduce the clinical relapses and magnetic resonance imaging lesions of MS.^[5]^

Multiple epidemiological characteristics of MS have been a research hotspot.^[6]^ According to previous literature, the prevalence, disability-adjusted life years (DALYs), incidence, and deaths of MS have significant regional and gender differences, but most previous studies were limited to specific regions.^[7]^ The epidemiological data of MS in different regions or different races are not perfect, and the epidemiological study of MS in low-income areas is more meaningful. According to reports from the World Health Organization or the Multiple Sclerosis International Federation, the global early diagnosis and prevalence characteristics of MS have not yet been fully studied, especially in resource-poor area; it is still of great significance to develop and popularize MS-related medical education based on international guidelines.^[8]^ In this context, Global Burden of Disease (GBD) database (the GBD database is a global health data resource that is widely used to analyze the changing trends of disease burdens) provides systematic data, and through the study of age, gender, or regional heterogeneity, it can further explore the changes of epidemiological characteristics of MS worldwide, which is particularly important for the research of etiology, disease prevention, diagnosis, and treatment of MS.^[9]^

Regarding the global GBD study on MS, the research by Khan and Hashim included the analysis and prediction of multiple regional and epidemiological factors, but it overlooked the crucial epidemiological factor of mortality. The study by Wallin et al in 2016 did not include a predictive model for the future epidemiological characteristics of MS. The research by Gombolay et al mainly focused on the age group of children. In our study, based on the GBD database, we analyzed the global multi-epidemiological data (prevalence, incidence, DALYs, and deaths) of MS patients from 1990 to 2021, and made a systematic evaluation based on sociodemographic index (SDI) and other social factors. In addition, we combined Bayesian models with multiple factors to predict the global burden of MS until 2050.^[10–12]^

## 2. Methods

The study followed the recommendations of the Guidelines for Accurate and Transparent Health Estimates Reporting. This study followed the GBD code for data use, and all individual data were anonymized in accordance with the ethical guidelines of the Declaration of Helsinki. All statistical analyses and data visualizations were performed using R (version 4.4.2) and JD_GBDR (V2.37, Jingding Medical Technology Co., Ltd.).

### 2.1. Data source and standardization

This study utilized the GBD database (1990–2021), systematically obtaining global epidemiological data for MS across 204 countries and regions. The dataset encompasses 2795 MS cases recorded in 2021, covering core metrics including incidence, prevalence, mortality, and DALYs. After data extraction, the original data were cleaned under a standardized quality control process, including elimination of abnormal data, multiple imputation of missing values (using linear regression and the neighborhood mean method), and age standardization according to the World Health Organization’s standard population structure to eliminate the interference of population aging; the GBD 2021 examined over 370 diseases and injuries in 204 countries and regions, providing detailed data on incidence, mortality, DALYs, and age-standardized rates (ASRs).^[12]^ All indicators were used as benchmark units per 100,000 population, ensuring the validity of comparisons across regions and across time.

### 2.2. Trend analysis and heterogeneity assessment

In this part of the study, piecewise regression model and Joinpoint regression analysis were used to quantify the long-term trends (time trends) of different epidemiological characteristics of MS in the world and across regions. The joint regression model was segmented linear Joinpoint regression (Joinpoint Regression Program 4.9.1.0, developed by the National Cancer Institute, Los Angeles), and the validity of the model hypothesis was verified by segmented point significance, residual normality, and goodness of model fit. To determine the location of the breakpoint and its validity, we used information standards such as Akaike information criterion and Bayesian information criterions for verification. Piecewise regression and Joinpoint regression models were used to analyze the long-term trends of epidemiological characteristics of MS in the world and different regions. All models are constructed based on the generalized addable mixture model framework, and the basic hypotheses of the models are verified by linear hypothesis, heteroskedasticity, autocorrelation, and over-discrete test. To control for potential confounders, such as gender, age, and socioeconomic status, we adjusted them during the analysis. Annual average percent change in age-standardized incidence rate (ASIR) was used to measure trend strength, and 95% confidence intervals were calculated using Monte Carlo simulations to verify the significance of differences and trend changes. Based on the SDI, the analysis of regional differences was divided into 5 levels: high, medium-high, medium, medium-low, and low. The temporal and spatial patterns of disease burden of MS in high-income and low-middle income areas were explored. In addition, for local hot spots in the world (e.g., Western Europe, Andean Latin America), the Moran *I* index was used to identify the characteristics of geographic clustering.

### 2.3. Sex-age interaction and health inequalities study

In this part, a sex-based generalized additive model was constructed to dynamically analyze the gender differences in various epidemiological indicators of MS. Considering the interaction between sex and age, we constructed a gender-based generalized additive model and analyzed the differences between genders within different age groups. Based on the above, age-specific analyses were conducted using 10-year age group as the benchmark, and chi-square tests were used to compare the differences in incidence, mortality, and DALYs distribution between different genders in different age groups. Health inequality assessment combined concentration index (CI) and Lorentz curve to quantify the gradient relationship between SDI level and disease indicators, and to identify the contribution of income, access to health care, education level, and other factors to health inequality through decomposition analysis.

### 2.4. Construction and validation of prediction model

The construction of epidemiological prediction models for MS was based on the Bayesian hierarchical regression framework, and the 3 covariates of time trend, population density prediction, and SDI development trajectory were integrated in the models. Model fit was evaluated by calculating the *R*^2^ value, residual analysis, and using the deviance information criterion method. For the Bayesian hierarchical regression model, we select a priori distribution such as fixed effect, random effect, and variance parameters, and verify its rationality by a posterior prediction test. Markov chain Monte Carlo algorithm was used to train the model. The prediction performance was evaluated by the set-aside method (1990–2010 was the training set, 2011–2021 was the validation set), and the prediction intervals of incidence, mortality, and other indicators in 2022 to 2050 were finally generated. Finally, the models were tested using sensitivity analyses that included the potential effects of changes in fertility rate, life expectancy, and diagnosis rate on the predictions; sensitivity analysis also includes checking the convergence of the Markov chain Monte Carlo algorithm through trace plots.

### 2.5. Statistical methods and visualization

The statistical analysis and visualization of this study were implemented in the R language (version 4.2.1). The key analysis parts included: first, nonlinear trend test, using segmented package to test the nonlinear trend of various epidemiological factors and confounding variables of MS; second, spatial analysis, which was a geographically weighted regression analysis by spdep package; third, health inequality analysis: the con-index package was used to calculate the CI of epidemiological factors and confounding variables of MS; fourth, prediction modeling: the INLA package was used to fit the Bayesian spatiotemporal model; fifth, purpose of the frontier analysis: to assess the “optimal” and “real” gap between the “optimal” and “real” burden of MS disease at different SDI levels. Model building: output-oriented model using data envelopment analysis with SDI as the input variable and DALYs rate as the output variable. Assuming variable return at scale, confidence intervals for efficiency scores were calculated by repeated sampling (1000 times) by Bootstrap. Verification steps: Kolmogorov–Smirnov test for efficiency score (according to Beta distribution). Sensitivity analysis: Efficiency ranking consistency between constant return at scale and variable return at scale models (Spearman ρ > 0.8). In the visualization part, Python Matplotlib (3.6.0 version) and Tableau (2022.3 version) were combined to realize the visual display of hierarchical heat map; biaxial clustering bar chart and dynamic trend curve according to the characteristics of multidimensional data, and on this basis, to ensure the intuitive presentation of complex spatial patterns and time trends.

## 3. Results

Table S1, Supplemental Digital Content, https://links.lww.com/MD/Q295 demonstrates that between 1990 and 2021, global MS incidence counts increased by 49.9% (41,970–62,920 cases per 100,000 population), while the ASIR declined by 3.46% (0.80–0.78 per 100,000), suggesting population growth drove absolute case increases despite a modest reduction in age-adjusted disease risk. Marked regional disparities emerged: ASIR rose in Western Europe (+27.77%) and Andean Latin America (+31.56%), whereas low-middle SDI areas experienced substantial case surges with smaller ASIR increases (e.g., South Asia: +122% cases vs +10.99% ASIR). Notable declines occurred in Central Asia (−9.52%) and Eastern Europe (−10.56%), while trends in high-income North America remained statistically inconclusive. These patterns highlight a shifting MS burden toward high-income regions and escalating public health challenges in prevention and management for low-to-middle-income populations.

Table S2, Supplemental Digital Content, https://links.lww.com/MD/Q295 reveals that global MS prevalence cases surged by 87.9% (1.0 to 1.9 million/100,000) from 1990 to 2021, while the ASIR remained stable (−0.42%), likely due to improved survival/diagnosis. High-SDI regions saw rising age-standardized prevalence rate (ASPR) (Western Europe: +34.10%; high SDI aggregate: +14.80%) alongside case growth. For consistency, unadjusted prevalence rates in 2021 were: Western Europe (127.91 cases/100,000), Central Latin America (130.50 cases/100,000), Andean Latin America (29.00 cases/100,000) (Table S1, Supplemental Digital Content, https://links.lww.com/MD/Q295). Extreme burden spikes occurred in Central Latin America (+56.92% ASIR) and Andean Latin America (+46.77% ASIR), while Central Asia (−0.85% ASIR) and Oceania (+0.58% ASIR) showed neutral trends. These patterns highlight widening disparities: high-income regions manage rising chronic prevalence, while low-resource settings face unmitigated case surges. Despite a global decline in age-standardized mortality (−12.76%) and DALY rates (−11.02%) for MS from 1990 to 2021, absolute deaths and DALYs surged by 79% and 69.5%, respectively, driven by population growth and prolonged disability. Stark disparities emerged: high-income North America saw mortality ASIR rise sharply (+40.40%), while Central/Eastern Europe achieved marked declines (−31.06% to −38.11%). These trends underscore a dual challenge: high-income regions struggle with chronic disease management, while resource-limited areas confront unmitigated case escalation amid diagnostic and therapeutic gaps (Tables S3 and S4, Supplemental Digital Content, https://links.lww.com/MD/Q295).

Age-standardized incidence, prevalence, and DALYs of MS in 204 countries and regions from 1990 to 2021 were analyzed. As shown in Table S5, Supplemental Digital Content, https://links.lww.com/MD/Q295, the global age-standardized prevalence increased by 8.88% (3.52–15.89) during this period, but the overall change in incidence was small (0.91% [−3.64 to 6.02]). Significant regional differences emerged: incidence increased by 33.99% in Canada, 30.07% in Australia, and 25.16% in the United Kingdom. In the United States, DALYs increased by 27.19% (0.62–56.10), while in the United Kingdom, DALY decreased by 18.41% (−29.87 to 6.49). These differences highlight the evolving burden of disease influenced by demographic, diagnostic, and environmental factors.

Table S2, Supplemental Digital Content, https://links.lww.com/MD/Q295 reports directly modeled global ASPR (22.17/100,000). Table S5, Supplemental Digital Content, https://links.lww.com/MD/Q295 provides country-level ASPR estimates; its implied global value (≈21.89/100,000) derives from population-weighted aggregation of 204 nations. This 1.3% difference falls within Table S2, Supplemental Digital Content, https://links.lww.com/MD/Q295, uncertainty interval (19.7–24.82), reflecting methodological variations in GBD’s hierarchical modeling approach.

**Figure 1.**
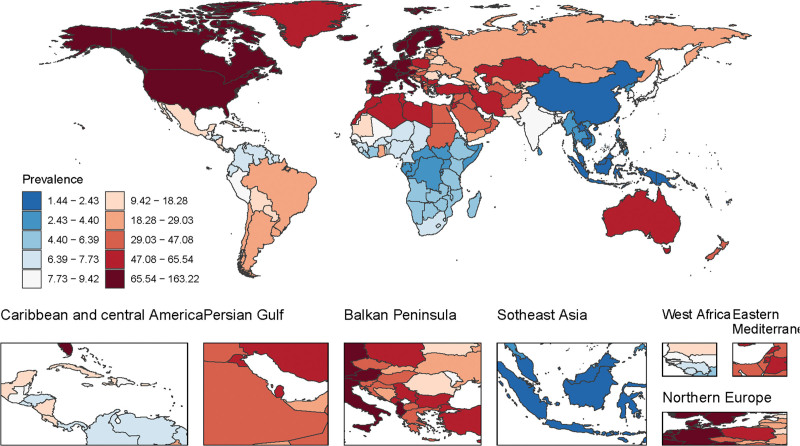
Age-standardized multiple sclerosis prevalence per 100,000 population in 2021 for both sexes, by location.

Regional variations in MS prevalence are evident, with standardized rates >60/100,000 in North America, Western Europe, and North Africa in 2021 (Fig. 1). Mortality and DALY rates were highest in North America, Northern Europe, and Australia (40–130 deaths/100,000; Figure S1, Supplemental Digital Content, https://links.lww.com/MD/Q294), while East Africa and Asia had the lowest rates (Figures S2 and S3, Supplemental Digital Content, https://links.lww.com/MD/Q294).

**Figure 2.**
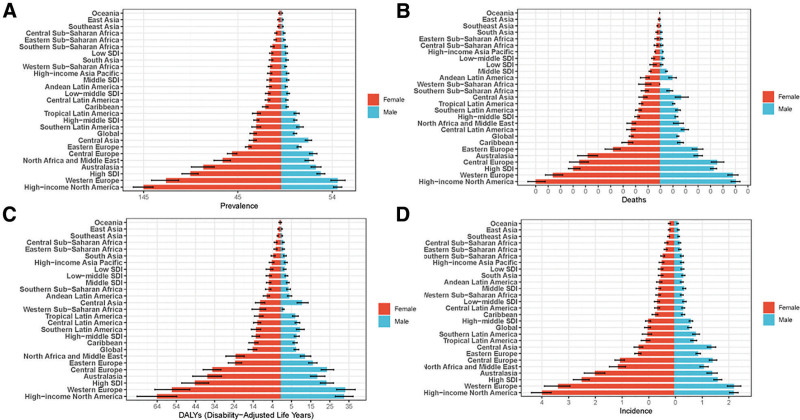
Regional differences in epidemiological characteristics of MS between men and women. Gender differences in the (A) prevalence of MS, (B) MS deaths, (C) DALYs of MS, and (D) incidence of MS. MS = multiple sclerosis.

**Figure 3.**
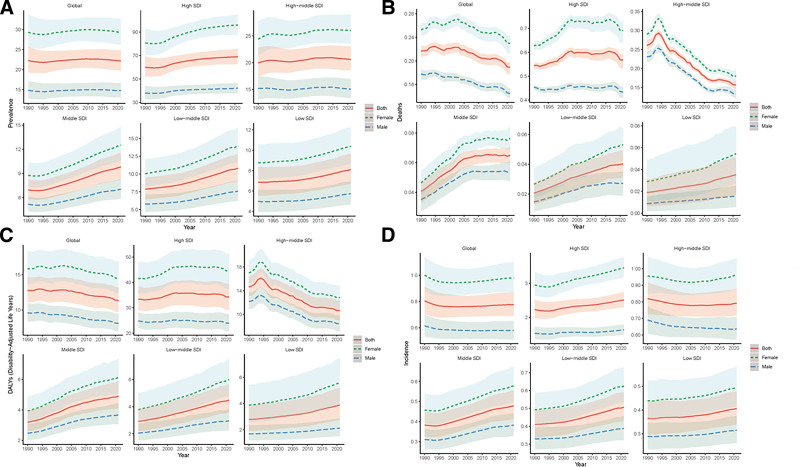
Regional time trends of each epidemiological feature of MS. (A) Temporal and regional trends in the prevalence of MS. Regional temporal trends in (B) MS deaths, (C) DALYs of MS, and (D) the incidence of MS. MS = multiple sclerosis.

Gender disparities showed higher MS burden in females across all metrics (Fig. 2). Notably, in Western Sub-Saharan Africa and the Caribbean, mortality and DALY gender gaps were wider in females (Fig. 2B and C). In order to comprehensively reflect the epidemiological trend of MS and the trend changes under various factors, we divided the analysis direction according to regions and investigated various epidemiological factors in the following study. As shown in Figure 3A, for the prevalence of MS, it showed an increasing trend in high SDI, medium SDI, medium to low SDI, and low SDI countries, and a stable trend in the world and high to medium SDI countries. As shown in Figure 3B, the trend of deaths of MS has different states, which decreases globally and in high-middle SDI countries, but increases in other SDI countries, similar to the DALYs of MS (Fig. 3C). In addition, there is the incidence of MS, which has leveled off globally, decreased in high-middle SDI countries, and increased significantly in other SDI countries (Fig. 3D).

**Figure 4.**
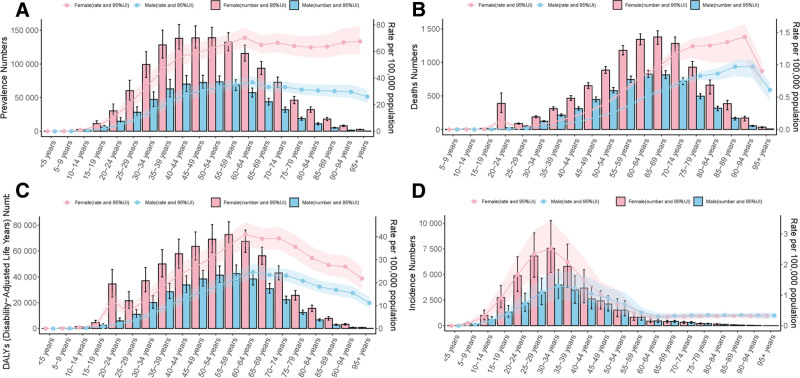
Two-coordinate clustered bar chart of MS for sex and age. Two-coordinate clustered bar chart of MS for sex and age in the (A) prevalence of MS, (B) MS deaths, (C) DALYs of MS, and (D) the incidence of MS. MS = multiple sclerosis.

In order to further explore the gender and age differentiation characteristics of each epidemiology of MS, we performed a 2-coordinate clustered bar chart of MS based on the obtained data. As shown in Figure 4A, the trend of MS prevalence was mostly concentrated between the ages of 35 and 65 years, and it was more significant in women than in men, and the DALYs of MS were similar. For the deaths of MS, most of them were between 55 and 75 years old, and more significantly in women than in men (Fig. 4B). As shown in Figure 4D, the incidence of MS tends to be younger, mostly between 20 and 40 years old. Similarly, it is more significant in women than in men.

**Figure 5.**
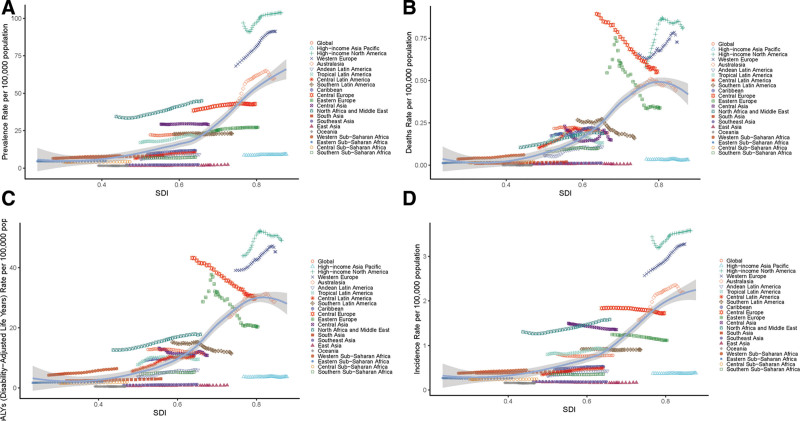
Standardized diagnostic rates of MS by SDI and SDI-based expected values from 1990 to 2021. The blue line represents the average expected relationship between SDI and MS diagnosis rates for 21 regions and globally for the estimation period 1990 to 2021. (A) Prevalence. (B) Deaths. (C) DALYs. (D) Incidence. DALYs = disability-adjusted life years, MS = multiple sclerosis, SDI = sociodemographic index.

In order to explore the relationship between age-standardized data and SDI in each GBD region of 21, annual time series from 1990 to 2016 were presented and Figure 5 was plotted. As shown in Figure 5A, in North America and Western Europe, the prevalence of MS increased substantially over time, particularly significantly compared to other stationary regions (*r* = 0.6975, *p* < 0.001). As shown in Figure 5B, MS deaths decreased significantly over time in most parts of Europe, but notably increased in North America and Western Europe (*r* = 0.6688, *p* < 0.001). Similarly, as shown in Figure 5C, MS DALYs have increased over time in high-income Western Europe and North America, but have declined in Central and Eastern Europe and are relatively flat in other regions (*r* = 0.6627, *p* < 0.001); the aforementioned regional differences in incidence rates and DALY are ASR. Finally, as shown in Figure 5D, while the incidence of MS showed a stable trend in most regions, high-income regions such as Western Europe and North America showed an upward trend over time (*r* = 0.6296, *p* < 0.001). In addition, we conducted an analysis of the changes in the gross disparities of the ASIR, ASPR, death rates, and DALYs rates for MS across all ages between the countries with the highest and lowest SDI from 1990 to 2021, as shown in Table 1.

**Table 1:** Changes in gross disparities of ASIR, ASPR, deaths, and DALYs rates for multiple sclerosis for all ages between the highest and lowest SDI countries, 1990 to 2021.

Indicator (per 100,000)	1990			2021		
	Coefficient	Lower CI	Upper CI	Coefficient	Lower CI	Upper CI
ASIR	1.03	0.78	1.28	1.39	1.11	1.68
ASPR	26.10	19.37	32.83	39.15	30.86	47.43
Deaths rate	0.35	0.27	0.43	0.39	0.32	0.47
DALYs rate	19.53	15.04	24.02	22.22	18.36	26.09

ASIR = age-standardized incidence rates, ASPR = age-standardized prevalence rates, CI = confidence intervals, DALYs = disability-adjusted life years, SDI = sociodemographic index.

**Figure 6.**
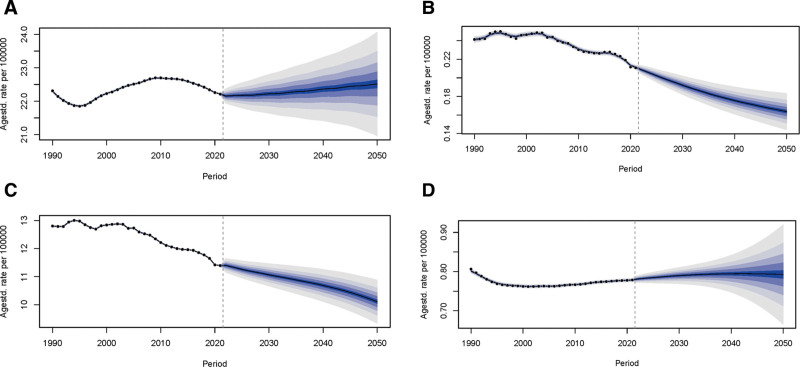
The proportion of each epidemiological factor of MS in all age groups was predicted until 2025. (A) Prevalence. (B) Deaths. (C) DALYs. (D) Incidence. DALYs = disability-adjusted life years, MS = multiple sclerosis, SDI = sociodemographic index.

Figure 6 presents trends in ASRs for MS from 1990 to 2050 (age-specific prediction plots are in Figures S4–S7, Supplemental Digital Content, https://links.lww.com/MD/Q294). Analysis of historical data (1990–2020) reveals: complex fluctuations in ASR prevalence, without evidence of stabilization within this period; a consistent decline in ASR deaths; a consistent decline in ASR DALYs; an initial decrease in ASR incidence followed by relative stability in later historical years. Projections (2020–2050) indicate: a potential stabilization of ASR prevalence from 2020 onward, although this projection relies heavily on the single transition point at 2020–2021 and warrants cautious interpretation regarding long-term stabilization; a continued, and potentially accelerating, decline in ASR deaths; a continued decline in ASR DALYs, though the rate of decrease may slow; a slow increase in ASR incidence. Furthermore, analysis of ASIR by SDI shows a distinct pattern: the highest ASIR values are consistently observed in high and high-middle SDI regions, while the lowest are found in low and low-middle SDI regions. Collectively, these historical trends reflect the influence of evolving diagnostics, therapies, and risk factors, while future projections suggest outcomes will depend on the interplay of medical advances, interventions, shifting risk factors, and persistent healthcare disparities, as evidenced by the SDI-related ASIR variations.

**Figure 7.**
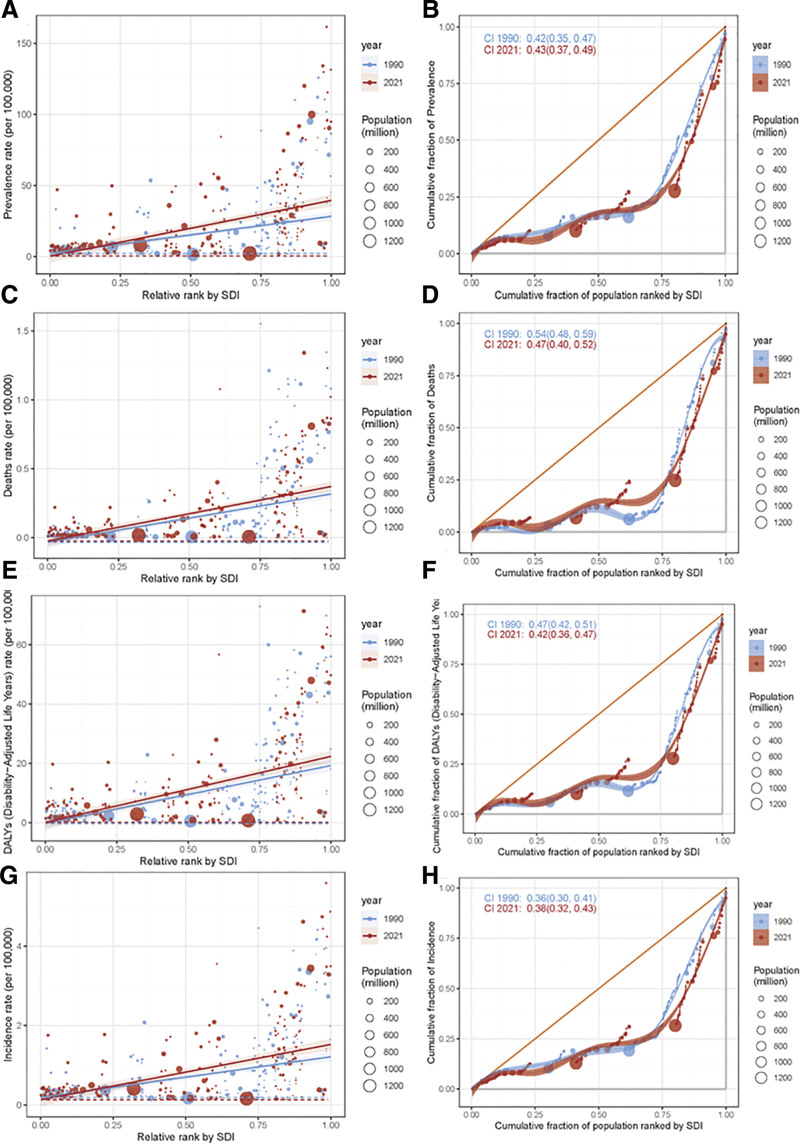
Changes in the distribution and inequality of health indicators based on sociodemographic index (SDI) from 1990 to 2021. SDI for the (A) prevalence of MS, (C) deaths of MS, (E) DALYs of MS, and (G) incidence of MS. CII for the (B) prevalence of MS, (D) deaths of MS, (F) DALYs of MS, and (H) deaths of MS. DALYs = disability-adjusted life years, MS = multiple sclerosis.

Similarly, based on the above research content, this study continued to analyze the changes of the epidemiological characteristics of MS based on the SDI from 1990 to 2021, including prevalence, mortality, DALYs rate, and incidence. As shown in Figure 7, scatter plots (A, C, E, G): the abscissa is the relative ranking by SDI, and the ordinate corresponds to the rates of different health indicators. Scatter points represent different regions or groups, and point size indicates population size. The distribution of each indicator was different between 2021 (red dots) and 1990 (blue dots). The trend line showed that the indicator rates changed with the relative ranking of SDI, and most indicator rates increased with the rise of SDI rank. Lorentz curve class plots (B, D, F, H): the abscissa is the cumulative proportion of the population ranked by SDI, and the ordinate is the cumulative proportion of the corresponding health indicator. The degree of deviation of the curve from the diagonal reflects the inequality of the distribution of health indicators, and the CI is given to quantify the inequality. Changes in CI values between 1990 (blue) and 2021 (red) indicate changes in inequalities in some health indicators.

**Figure 8.**
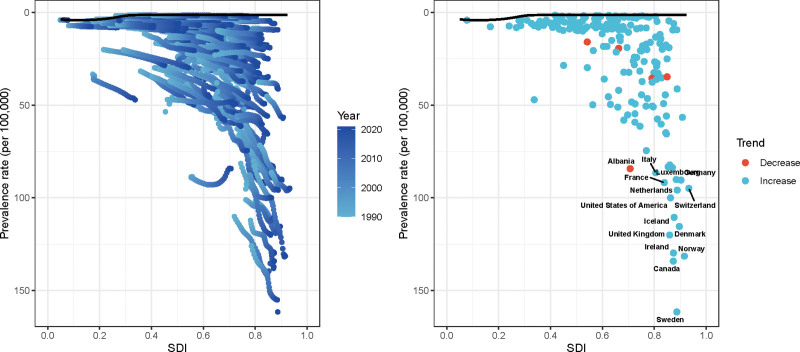
Frontier analysis of the relationship between different sociodemographic indices (SDI) and prevalence. Left panel: data are shown as lines, with colors representing different years (from 1990 to 2020); Right panel: data are presented as scatter points; blue scatter points indicate areas with increasing prevalence and red scatter points indicate areas with decreasing prevalence.

In addition, we also conducted frontier analysis of various epidemiological factors of MS. As shown in Figure 8, overall, the prevalence of MS has a downward trend with the increase of SDI, and the prevalence of most countries has an increasing trend (blue scatter points), and a few countries such as Albania and France have a downward trend (red scatter points).

As shown in Figure S8, Supplemental Digital Content, https://links.lww.com/MD/Q294, the study on the deaths of MS showed that, on the whole, with the increase of SDI, the prevalence of MS showed a decreasing trend in Albania, Norway, Denmark, and other countries, while the United Kingdom showed an increasing trend. As for DALYs, as shown in Figure S9, Supplemental Digital Content, https://links.lww.com/MD/Q294, the overall situation is similar to that mentioned above, but there is a downward trend in countries such as Denmark and Albania, and an increasing trend in countries such as the United Kingdom and Sweden. Finally, the incidence of MS, the overall situation is basically consistent with the first 3; the incidence of most countries shows an upward trend with the growth of SDI; it is worth noting that the incidence of Netherland shows a downward trend (Figure S10, Supplemental Digital Content, https://links.lww.com/MD/Q294).

## 4. Discussion

Based on the GBD database, this study systematically evaluated the evolution of MS epidemiological characteristics from 1990 to 2021, and revealed the dynamic changes of the disease burden of MS across different demographic characteristics, socioeconomic differences, and wide geographic areas. The results showed that although ASIR of MS showed a slight downward trend (−3.46%), the number of MS cases showed a significant upward trend (49.9%) due to population growth, notably in low and middle SDI regions. The results show that the epidemiological model of MS has changed from a simple risk of disease to a complex public health problem closely related to medical resource allocation and population structure.

This study found that between 1990 and 2021, the number of MS cases increased by 87.9% (1.0–1.9 million per 100,000), while the ASPR decreased by only 0.42%. The results suggested that population growth and longer survival time were the main factors causing the increase of global burden of MS. Similar trends are also similar to those reported in the literature: for example, the spread of advanced early diagnostic techniques and widespread use of DMTs in high SDI countries such as North America and Western Europe have significantly improved the short-term or long-term quality of life of MS patients, but this is also one of the factors leading to the aging of the cumulative affected population.^[6,13,14]^ On the contrary, regions with low and medium SDI, such as Latin America and Sub-Saharan Africa, showed a paradoxical phenomenon that the number of MS cases increased rapidly but the increase of ASPR was not significant.^[15,16]^ This phenomenon may reflect 2 issues. First, actual risk factors for MS (e.g., rising rates of obesity and reduced exposure to ultraviolet light) are on the rise in these regions.^[17,18]^ Secondly, a large number of MS cases have not been accurately diagnosed due to the backward level of disease diagnosis, which is also supported by the high rate of misdiagnosis of MS in Africa.^[19]^

Regional analysis further revealed the “bimodal differentiation” of disease burden. In regions with high SDI (e.g., North America and Western Europe), the ASPR increased significantly (+34.10%), while in regions with low and medium SDI, the ASPR increased slightly (+41.22%), but the number of cases increased sharply by 184%, accompanied by an abnormal increase in age-standardized mortality rate (ASMR) (e.g., Central Latin America +110.91%). This divergence suggests that high-income countries have entered the “chronic disease management stage,” with increasing demands for long-term care and rehabilitation. However, the “diagnosis-treatment gap” still exists in low-resource areas, leading to increased disability progression and premature mortality.^[20–23]^ This is also consistent with the results of the global pattern of inequalities in neurological health identified in the GBD report on neurological diseases 1990 to 2016.^[9,24]^

In addition, this study confirmed that there are significant gender differences in MS incidence and disease burden, with females having a higher prevalence (peak at 35–65 years of age), mortality (peak at 55–75 years of age), and DALYs. This result may be related to a complex interaction between environment and biology.^[25,26]^ Firstly, the increase of smoking rate and vitamin D deficiency in women aggravate the imbalance of immune regulation.^[27–29]^ Second, obesity may promote neuroinflammation.^[30,31]^ Hormone replacement therapy has also been suggested to alter the disease course of MS.^[32]^ Of note, the increase in mortality was greater among women in low-SDI regions (e.g., 315% increase in DALYs in West Africa), suggesting that differences in health care resources may be a major contributor, a finding that calls for prioritizing sex-specific MS screening programs in resource-limited regions.

This study also found that changes in many epidemiological characteristics of MS are becoming less related to geographical differences, with the increase of ASPR slowing down in high latitudes, while the prevalence of MS increased significantly in low and middle latitudes (e.g., northern Australia and Panama). This result may reflect 2 changes. First, the role of traditional latitude-related factors such as UV exposure and vitamin D levels is being weakened by increasing urbanization.^[33,34]^ Secondly, the change of dietary structure (such as the increase of processed food intake) and new risk factors such as intestinal flora disorder under the background of globalization have regional differences, which are also one of the reasons for the change of epidemiological factors of MS.^[35–38]^ It is worth noting that there is an “inverse gradient” phenomenon within regions with high SDI, such as North America (e.g., the prevalence of 266.9 per 100 000 in Canada), which suggests that attention should be paid to the differential effects of regional environmental toxins (such as industrial pollutants) or the clustering of genetically susceptible populations.^[39–41]^

Based on the Bayesian model, it can be known that these historical trends reflect the influence of evolving diagnostics, therapies, and risk factors, while future projections suggest outcomes will depend on the interplay of medical advances, interventions, shifting risk factors, and persistent healthcare disparities, as evidenced by the SDI-related ASIR variations. Furthermore, other studies on MS in the GBD database, such as Khan and Hashim (2025), analyzed multiple regions and epidemiological factors, but did not include mortality analysis; Wallin et al (2019)’s research lacked a predictive model for the future epidemiological characteristics of MS; Gombolay et al (2025) mainly focused on the pediatric population. This study is based on the GBD database and not only systematically analyzed various epidemiological data (prevalence, incidence, DALYs, and mortality) of MS worldwide from 1990 to 2021, but also conducted stratified assessment based on social factors such as SDI, and combined Bayesian models and multiple factors to predict the global burden of MS by 2050, filling the gaps in the above studies.^[10–12]^

Based on the findings of this study, we should first focus on improving the full-cycle management of MS patients in areas with high SDI, including vocational rehabilitation, cognitive intervention, and comorbidity prevention and control.^[42–46]^ In order to increase the diagnosis rate of MS and other related diseases for early intervention, it is necessary to strengthen the professional training of specialists in primary medical institutions and promote low-cost diagnostic tools in low and medium SDI areas.^[47–49]^ Thirdly, given that the burden of MS increases among women of childbearing age (20–40 years old), it is recommended to enhance monitoring of maternal immunity and hormones. The main reason is that the dynamic changes in immunity during pregnancy/postpartum (such as a 20%–40% increase in the risk of recurrence after childbirth) and hormonal fluctuations (such as estrogen withdrawal) significantly affect disease activity, pregnancy outcomes, and treatment management (DMTs), and individualized intervention is necessary to reduce the risk of complications and disability.^[50–52]^

## 5. Limitations

Our study may have several limitations. First, we used multiple imputation to missing raw data in some regions, which may lead to overestimation (e.g., assuming a linear relationship between diagnosis and SDI) or underestimation (e.g., not accounting for cases treated by traditional medicine) of model performance. Secondly, the medium- and long-term impacts of the COVID-19 pandemic on the MS diagnosis and treatment system have not been systematically evaluated in the models of this study.

## 6. Conclusion

This study revealed the evolution and regional and gender heterogeneity of the global burden of MS from 1990 to 2021. It was found that although the survival of MS patients has been prolonged through the progress of diagnosis and treatment technology, the high SDI regions still face the complex challenge of managing aging patients. However, the mortality of MS patients in low SDI regions is still high due to the lack of diagnostic ability and medical resources. In addition, the burden of prevalence, mortality and DALYs of MS in females was heavier, and the gender inequality was more obvious in low-income areas. It is suggested that in the future, it is necessary to further optimize the full-cycle health management for MS patients in high SDI areas, strengthen the early screening system of MS in low- and middle-income countries, and narrow the gap in access to treatment through international cooperation to cope with the global differentiation of MS burden.

## Acknowledgments

This study was generously supported by Jingding Medical Tech, to whom we extend our sincere gratitude. We especially thank them for providing authorization and technical support for the JD_GBDR software. The team at Jingding Medical Tech offered invaluable assistance in data processing.

## Author contributions

**Conceptualization:** Jie Liu.

**Data curation:** Yuezhen Shen, Jie Liu.

**Formal analysis:** Yuezhen Shen, Jie Liu, Huasheng Fan, Peijun Han.

**Funding acquisition:** Jie Liu, Huasheng Fan.

**Investigation:** Jie Liu, Huasheng Fan, Han Wang, Yuchen Guo.

**Methodology:** Huasheng Fan, Han Wang, Yinfu Xu, Yuchen Guo.

**Project administration:** Han Wang, Yuchen Guo.

**Resources:** Han Wang, Yinfu Xu, Peijun Han.

**Software:** Yinfu Xu, Peijun Han.

**Supervision:** Yinfu Xu, Yuchen Guo, Peijun Han.

**Writing – original draft:** Yuchen Guo.

**Writing – review & editing:** Yuchen Guo.

## Supplementary Material




