# Supplementary material for: Systematic study and 30-year projections of global and multi-regional burden of multiple sclerosis, 1990–2021
Source: Medicine (Baltimore). 2025 Oct 31;104(44):e45089. doi: 10.1097/MD.0000000000045089 (PMC12582701; doi:10.1097/MD.0000000000045089)
Supplement: Supplementary file 1 [file medi-104-e45089-s001.docx]

Table S1 The incidence cases and age-standardized incidence rate of multiple sclerosis in 1990 and 2021, and its temporal trends from 1990 to 2021 ASR Age-standardized rate

| Characteristics | 1990 |  |  | 2021 |  | 1990-2021 |
| --- | --- | --- | --- | --- | --- | --- |
|  | Incidence cases No.x10'5 (95% UI) | ASR per 100,000 No. (95% UI) |  | Incidence cases No.x10'5 (95% UI) | ASR per 100,000 No. (95% UI) | Percentage change in age-standardised rates |
| Global | 41970(36606,48235) | 0.80(0.70,0.91) |  | 62920(56015,70635) | 0.78(0.69,0.87) | -3.46(-5.52,-0.69) |
| High SDl | 21063(18609,23755) | 2.23(1.98,2.52) |  | 25926(23686,28293) | 2.51(2.30,2.74) | 12.66(7.96,18.45) |
| High-middle SDl | 9143(8123,10318) | 0.82(0.72,0.92) |  | 10507(9447,11562) | 0.79(0.72,0.88) | -3.40(-7.00,0.27) |
| Middle SDl | 6145(5141,7333) | 0.38(0.33,0.45) |  | 12594(10823,14531) | 0.48(0.41,0.55) | 25.08(20.17,30.38) |
| Low-middle SDl | 4107(3358,4995) | 0.41(0.34,0.49) |  | 9858(8341,11614) | 0.51(0.43,0.59) | 23.52(17.72,29.12) |
| Low SDI | 1455(1184,1792) | 0.37(0.30,0.44) |  | 3974(3311,4754) | 0.41(0.34,0.48) | 11.15(7.03,15.43) |
| Andean Latin America | 103(83,126) | 0.32(0.27,0.39) |  | 290(239,346) | 0.43(0.35,0.50) | 31.56(25.17,38.57) |
| Australasia | 344(306,387) | 1.57(1.40,1.77) |  | 674(592,761) | 2.19(1.92,2.47) | 39.26(28.78,50.39) |
| Caribbean | 157(130,188) | 0.46(0.38,0.55) |  | 260(220,302) | 0.52(0.44,0.61) | 13.29(8.26,18.04) |
| Central Asia | 832(735,936) | 1.50(1.32,1.68) |  | 1346(1200,1504) | 1.36(1.21,1.51) | -9.52(-13.32,-4.93) |
| Central Europe | 2371(2134,2645) | 1.84(1.65,2.05) |  | 1842(1677,2017) | 1.72(1.58,1.89) | -6.03(-9.01,-2.33) |
| Central Latin America | 490(398,598) | 0.35(0.29,0.42) |  | 1305(1113,1524) | 0.49(0.42,0.57) | 40.01(31.93,48.67) |
| Central Sub-Saharan Africa | 102(81,128) | 0.24(0.19,0.29) |  | 279(224,347) | 0.24(0.20,0.30) | 2.15(-1.43,5.93) |
| East Asia | 2054(1624,2547) | 0.18(0.14,0.22) |  | 2937(2373,3505) | 0.17(0.14,0.20) | -7.48(-11.56,-2.99) |
| Eastern Europe | 2937(2545,3338) | 1.25(1.09,1.42) |  | 2127(1919,2349) | 1.11(1.02,1.22) | -10.56(-16.27,-3.73) |
| Eastern Sub-Saharan Africa | 385(308,486) | 0.27(0.22,0.33) |  | 946(766,1173) | 0.26(0.22,0.32) | -1.31(-4.23,1.36) |
| High-income Asia Pacifc | 703(569,845) | 0.37(0.30,0.45) |  | 734(604,873) | 0.38(0.31,0.46) | 1.51(-1.99,4.57) |
| High-income North America | 10544(9263,12009) | 3.44(3.04,3.91) |  | 12296(11438,13265) | 3.58(3.33,3.86) | 4.04(-2.99,12.18) |
| North Africa and Middle East | 4098(3545,4720) | 1.31(1.15,1.50) |  | 10579(9170,12215) | 1.59(1.38,1.83) | 21.27(17.04,25.27) |
| Oceania | 8(6,11) | 0.16(0.13,0.20) |  | 18(14,23) | 0.15(0.12,0.19) | -5.61(-8.98,-2.24) |
| South Asia | 3664(2991,4483) | 0.38(0.31,0.45) |  | 8133(6736,9691) | 0.42(0.35,0.50) | 10.99(6.71,15.27) |
| Southeast Asia | 741(597,936) | 0.19(0.15,0.23) |  | 1348(1096,1616) | 0.19(0.15,0.22) | -1.62(-4.28,0.98) |
| Southern Latin America | 449(382,526) | 0.92(0.78,1.08) |  | 643(548,765) | 0.90(0.77,1.08) | -1.67(-5.75,2.91) |
| Southern Sub-Saharan Africa | 161(131,199) | 0.35(0.29,0.42) |  | 292(239,351) | 0.36(0.29,0.42) | 1.59(-1.98,5.51) |
| Tropical Latin America | 1091(918,1291) | 0.80(0.67,0.92) |  | 2302(1950,2656) | 0.90(0.77,1.04) | 12.83(7.43,18.95) |
| Western Europe | 10169(9002,11339) | 2.57(2.28,2.88) |  | 12791(11476,14202) | 3.28(2.93,3.66) | 27.77(23.54,32.44) |
| Western Sub-Saharan Africa | 566(464,691) | 0.38(0.31,0.45) |  | 1777(1513,2099) | 0.45(0.38,0.52) | 18.80(14.26,24.03) |

**Table S2** The prevalence cases and age-standardized incidence rate of multiple sclerosis in 1990 and 2021, and its temporal trends from 1990 to 2021 ASR Age-standardized rate

| Characteristics | 1990 |  |  | 2021 |  | 1990-2021 |
| --- | --- | --- | --- | --- | --- | --- |
|  | Prevalence cases No.x10'5 (95% UI) | ASR per 100,000 No. (95% UI) |  | Prevalence cases No.x10'5 (95% UI) | ASR per 100,000 No. (95% UI) | Percentage change in age-standardised rates |
| Global | 1004660(868374,1165225) | 22.26(19.30,25.65) |  | 1887768(1688654,2113708) | 22.17(19.77,24.82) | -0.42(-3.59,3.25) |
| High SDl | 606098(526358,696786) | 60.06(52.18,69.32) |  | 1008732(915744,1106678) | 68.95(62.65,75.60) | 14.80(8.60,21.32) |
| High-middle SDl | 212017(185100,241847) | 19.96(17.44,22.82) |  | 349780(314375,391422) | 20.59(18.51,22.98) | 3.15(-0.16,7.11) |
| Middle SDl | 96122(79227,115686) | 6.95(5.80,8.26) |  | 272695(230740,321521) | 9.81(8.28,11.55) | 41.22(36.59,45.89) |
| Low-middle SDl | 66521(54173,81538) | 7.87(6.42,9.52) |  | 190909(157902,228172) | 10.80(8.98,12.85) | 37.29(31.38,43.66) |
| Low SDI | 22611(18205,27916) | 6.87(5.54,8.39) |  | 63727(51976,77021) | 8.10(6.68,9.66) | 17.90(13.75,22.24) |
| Andean Latin America | 1659(1321,2082) | 6.24(5.01,7.72) |  | 5908(4856,7125) | 9.15(7.55,11.01) | 46.77(40.47,54.33) |
| Australasia | 8989(7924,10221) | 40.31(35.56,45.85) |  | 23860(20717,27287) | 59.95(51.99,68.82) | 48.73(36.28,61.87) |
| Caribbean | 2777(2261,3351) | 9.34(7.67,11.18) |  | 6061(5054,7141) | 11.69(9.73,13.80) | 25.07(18.79,31.27) |
| Central Asia | 15247(13423,17147) | 29.34(25.80,33.07) |  | 27048(23998,30485) | 29.09(25.82,32.72) | -0.85(-4.91,4.04) |
| Central Europe | 54038(47370,61361) | 38.47(33.70,43.70) |  | 64266(58577,70830) | 42.85(38.65,47.32) | 11.39(6.98,16.87) |
| Central Latin America | 7599(6068,9434) | 6.60(5.37,8.08) |  | 27545(22687,32605) | 10.36(8.55,12.25) | 56.92(47.84,66.50) |
| Central Sub-Saharan Africa | 1433(1111,1838) | 4.01(3.10,5.01) |  | 4117(3260,5247) | 4.34(3.48,5.44) | 8.34(3.84,13.03) |
| East Asia | 18985(14299,25029) | 1.59(1.23,2.06) |  | 43124(34473,53508) | 2.31(1.81,2.92) | 45.19(38.72,52.58) |
| Eastern Europe | 60773(51652,70900) | 23.52(19.99,27.36) |  | 72076(66148,79156) | 27.24(24.96,29.87) | 15.82(6.61,28.07) |
| Eastern Sub-Saharan Africa | 5372(4195,6899) | 4.54(3.56,5.66) |  | 13907(10975,17620) | 4.80(3.83,5.91) | 5.64(2.93,8.67) |
| High-income Asia Pacifc | 17392(14170,21302) | 8.65(7.03,10.60) |  | 25966(21348,31155) | 9.24(7.57,11.21) | 6.89(3.39,10.33) |
| High-income North America | 307019(267198,354750) | 96.91(84.33,112.08) |  | 487309(453754,522560) | 103.61(96.44,111.33) | 6.91(-1.71,16.33) |
| North Africa and Middle East | 79893(68886,92261) | 34.48(29.91,39.62) |  | 266776(229600,309730) | 45.05(39.00,51.99) | 30.65(26.96,34.51) |
| Oceania | 82(60,111) | 1.60(1.21,2.07) |  | 197(146,264) | 1.61(1.21,2.09) | 0.58(-2.58,4.02) |
| South Asia | 58711(47246,72668) | 7.05(5.70,8.65) |  | 150822(124040,182276) | 8.39(6.87,10.08) | 18.89(14.41,23.38) |
| Southeast Asia | 8420(6353,11145) | 2.18(1.68,2.80) |  | 18128(14104,22960) | 2.40(1.87,3.04) | 9.96(6.86,13.21) |
| Southern Latin America | 10747(8945,12655) | 22.72(18.92,26.77) |  | 17955(14898,21352) | 23.05(19.03,27.43) | 1.46(-3.18,6.07) |
| Southern Sub-Saharan Africa | 2580(2060,3196) | 6.85(5.53,8.43) |  | 5448(4457,6656) | 7.29(6.00,8.78) | 6.44(2.75,10.47) |
| Tropical Latin America | 19438(16182,23189) | 17.08(14.35,20.20) |  | 55713(47373,65515) | 21.23(18.09,24.98) | 24.30(19.07,30.56) |
| Western Europe | 315331(274881,361434) | 68.13(59.33,78.37) |  | 544964(485454,613732) | 91.36(80.90,102.99) | 34.10(29.48,39.95) |
| Western Sub-Saharan Africa | 8176(6612,10124) | 6.49(5.27,7.92) |  | 26577(22149,31989) | 8.35(7.02,9.90) | 28.60(23.79,35.31) |

**Table S3** The deaths cases and age-standardized incidence rate of multiple sclerosis in 1990 and 2021, and its temporal trends from 1990 to 2021 ASR Age-standardized rate

| Characteristics | 1990 |  |  | 2021 |  | 1990-2021 |
| --- | --- | --- | --- | --- | --- | --- |
|  | Deaths cases No.x10'5 (95% UI) | ASR per 100,000 No. (95% UI) |  | Deaths cases No.x10'5 (95% UI) | ASR per 100,000 No. (95% UI) | Percentage change in age-standardised rates |
| Global | 9108(8711,9469) | 0.22(0.21,0.23) |  | 16302(15357,17040) | 0.19(0.18,0.20) | -12.76(-17.54,-8.34) |
| High SDl | 5721(5552,5853) | 0.54(0.53,0.56) |  | 10607(9817,11111) | 0.57(0.53,0.59) | 4.27(-1.19,9.06) |
| High-middle SDl | 2674(2556,2821) | 0.26(0.25,0.28) |  | 2933(2735,3114) | 0.16(0.15,0.17) | -39.80(-44.76,-34.46) |
| Middle SDl | 466(376,544) | 0.04(0.03,0.05) |  | 1752(1622,1912) | 0.07(0.06,0.07) | 59.97(33.77,96.51) |
| Low-middle SDl | 152(96,206) | 0.02(0.01,0.03) |  | 670(523,821) | 0.04(0.03,0.05) | 94.70(53.53,177.94) |
| Low SDI | 70(25,114) | 0.02(0.01,0.03) |  | 316(153,462) | 0.04(0.02,0.05) | 85.79(41.02,212.05) |
| Andean Latin America | 14(11,17) | 0.06(0.05,0.07) |  | 66(50,86) | 0.11(0.08,0.14) | 84.00(38.69,141.90) |
| Australasia | 98(89,108) | 0.43(0.39,0.47) |  | 214(186,243) | 0.45(0.39,0.50) | 5.46(-10.95,21.60) |
| Caribbean | 49(46,54) | 0.17(0.16,0.19) |  | 112(97,129) | 0.21(0.18,0.24) | 21.86(5.10,40.69) |
| Central Asia | 101(89,115) | 0.23(0.20,0.26) |  | 104(79,124) | 0.15(0.11,0.18) | -35.61(-50.94,-20.14) |
| Central Europe | 1301(1233,1383) | 0.90(0.85,0.95) |  | 1041(946,1146) | 0.55(0.50,0.61) | -38.11(-44.97,-31.23) |
| Central Latin America | 105(101,108) | 0.10(0.10,0.11) |  | 562(500,635) | 0.22(0.19,0.24) | 110.91(84.68,140.07) |
| Central Sub-Saharan Africa | 4(1,6) | 0.01(0.01,0.02) |  | 15(7,24) | 0.02(0.01,0.03) | 62.23(4.51,159.62) |
| East Asia | 50(29,74) | 0.01(0.00,0.01) |  | 117(92,146) | 0.01(0.00,0.01) | 13.35(-30.60,92.02) |
| Eastern Europe | 1393(1341,1451) | 0.55(0.53,0.57) |  | 997(889,1110) | 0.34(0.30,0.38) | -37.87(-45.31,-29.75) |
| Eastern Sub-Saharan Africa | 11(3,19) | 0.01(0.00,0.02) |  | 44(18,67) | 0.02(0.01,0.03) | 65.85(18.21,178.20) |
| High-income Asia Pacifc | 71(67,74) | 0.03(0.03,0.04) |  | 107(97,115) | 0.03(0.03,0.03) | -11.14(-17.03,-5.16) |
| High-income North America | 1867(1787,1941) | 0.58(0.56,0.60) |  | 4980(4642,5269) | 0.81(0.76,0.86) | 40.40(31.67,49.23) |
| North Africa and Middle East | 215(115,316) | 0.10(0.05,0.15) |  | 1005(829,1175) | 0.19(0.15,0.22) | 80.04(24.62,214.39) |
| Oceania | 0(0,0) | 0.00(0.00,0.00) |  | 0(0,0) | 0.00(0.00,0.00) | 25.57(-11.87,89.12) |
| South Asia | 68(27,114) | 0.01(0.00,0.02) |  | 298(195,395) | 0.02(0.01,0.02) | 85.59(24.86,286.35) |
| Southeast Asia | 24(16,32) | 0.01(0.00,0.01) |  | 90(75,103) | 0.01(0.01,0.01) | 76.41(30.93,173.40) |
| Southern Latin America | 125(118,132) | 0.27(0.25,0.28) |  | 131(118,144) | 0.16(0.14,0.17) | -40.40(-47.18,-33.65) |
| Southern Sub-Saharan Africa | 24(17,30) | 0.08(0.05,0.10) |  | 65(51,80) | 0.10(0.08,0.12) | 29.74(3.95,75.68) |
| Tropical Latin America | 104(99,109) | 0.11(0.10,0.12) |  | 340(312,364) | 0.13(0.12,0.14) | 20.88(11.09,31.20) |
| Western Europe | 3408(3294,3522) | 0.66(0.64,0.68) |  | 5668(5225,6004) | 0.73(0.68,0.77) | 10.53(3.72,17.11) |
| Western Sub-Saharan Africa | 75(40,114) | 0.04(0.02,0.05) |  | 343(210,499) | 0.06(0.04,0.09) | 71.52(5.61,203.65) |

**Table S4** The dalys cases and age-standardized incidence rate of multiple sclerosis in 1990 and 2021, and its temporal trends from 1990 to 2021 ASR Age-standardized rate

| Characteristics | 1990 |  |  | 2021 |  | 1990-2021 |
| --- | --- | --- | --- | --- | --- | --- |
|  | DALYs cases No.x10'5 (95% UI) | ASR per 100,000 No. (95% UI) |  | DALYs cases No.x10'5 (95% UI) | ASR per 100,000 No. (95% UI) | Percentage change in age-standardised rates |
| Global | 574234(496156,662160) | 12.78(11.10,14.72) |  | 973298(838210,1133291) | 11.37(9.77,13.23) | -11.02(-14.02,-8.03) |
| High SDl | 338312(294504,388986) | 33.39(29.00,38.42) |  | 535992(464951,609039) | 34.25(29.21,39.34) | 2.57(-0.86,6.22) |
| High-middle SDl | 155253(138829,174526) | 14.63(13.10,16.44) |  | 184675(158508,212439) | 10.66(9.08,12.35) | -27.16(-32.46,-22.68) |
| Middle SDl | 44213(35934,55135) | 3.19(2.59,3.98) |  | 135633(113504,163934) | 4.91(4.11,5.92) | 53.98(41.29,69.84) |
| Low-middle SDl | 25107(18942,33165) | 2.91(2.18,3.83) |  | 80709(64530,102168) | 4.48(3.59,5.66) | 54.10(40.11,72.10) |
| Low SDI | 10093(6720,14002) | 2.78(1.91,3.84) |  | 35016(24079,47214) | 3.88(2.78,5.17) | 39.40(23.86,60.04) |
| Andean Latin America | 965(783,1208) | 3.66(2.99,4.56) |  | 3808(3038,4678) | 5.94(4.75,7.29) | 62.08(38.30,89.47) |
| Australasia | 5269(4493,6109) | 23.55(20.08,27.39) |  | 11883(9897,14138) | 28.79(23.72,34.38) | 22.25(9.47,36.61) |
| Caribbean | 2714(2448,3090) | 9.08(8.18,10.37) |  | 5578(4849,6540) | 10.71(9.30,12.59) | 17.92(5.53,30.99) |
| Central Asia | 6816(5619,8141) | 13.20(10.90,15.73) |  | 9793(7529,12504) | 10.79(8.37,13.66) | -18.29(-27.88,-9.75) |
| Central Europe | 61483(56973,66792) | 43.30(40.05,47.13) |  | 48472(43059,54310) | 29.85(26.30,33.74) | -31.06(-36.68,-25.49) |
| Central Latin America | 6386(5703,7246) | 5.49(4.90,6.24) |  | 27925(24430,31706) | 10.50(9.19,11.92) | 91.21(71.77,111.57) |
| Central Sub-Saharan Africa | 545(388,771) | 1.57(1.11,2.19) |  | 1763(1278,2385) | 1.94(1.38,2.62) | 23.29(5.23,43.32) |
| East Asia | 7416(5385,10346) | 0.63(0.47,0.89) |  | 16096(11851,20994) | 0.86(0.63,1.13) | 35.73(14.24,55.69) |
| Eastern Europe | 72546(67132,78622) | 28.69(26.60,31.09) |  | 55278(48253,61669) | 20.41(17.70,22.84) | -28.85(-36.13,-21.82) |
| Eastern Sub-Saharan Africa | 1965(1367,2802) | 1.69(1.16,2.38) |  | 5718(3953,7772) | 2.03(1.40,2.73) | 19.82(8.20,35.72) |
| High-income Asia Pacifc | 7363(5775,9391) | 3.67(2.88,4.67) |  | 10243(7906,13248) | 3.66(2.80,4.71) | -0.32(-3.82,2.74) |
| High-income North America | 138072(115210,163388) | 43.96(36.73,51.91) |  | 250615(215531,285072) | 49.15(41.84,56.53) | 11.81(5.63,18.52) |
| North Africa and Middle East | 29651(22889,38837) | 12.62(9.74,16.53) |  | 106682(86362,130200) | 17.85(14.50,21.74) | 41.53(24.04,63.34) |
| Oceania | 23(15,34) | 0.45(0.29,0.66) |  | 56(36,81) | 0.46(0.30,0.66) | 0.67(-2.57,4.27) |
| South Asia | 19219(13756,26352) | 2.33(1.67,3.18) |  | 53446(39995,70343) | 2.99(2.23,3.92) | 28.41(18.47,39.16) |
| Southeast Asia | 3510(2668,4689) | 0.90(0.68,1.20) |  | 8811(6980,10909) | 1.18(0.94,1.45) | 30.75(16.55,49.40) |
| Southern Latin America | 7186(6295,8336) | 15.21(13.33,17.66) |  | 8975(7392,10755) | 11.44(9.39,13.71) | -24.79(-31.99,-17.82) |
| Southern Sub-Saharan Africa | 1639(1289,2068) | 4.59(3.57,5.72) |  | 3757(3074,4561) | 5.27(4.34,6.36) | 14.90(-0.43,34.10) |
| Tropical Latin America | 8766(7150,10783) | 7.72(6.28,9.49) |  | 24995(20489,30261) | 9.54(7.82,11.54) | 23.65(17.25,31.43) |
| Western Europe | 185325(161816,211811) | 39.12(33.88,44.82) |  | 288773(248772,330321) | 45.33(38.18,52.08) | 15.86(10.51,20.42) |
| Western Sub-Saharan Africa | 7377(4795,10164) | 4.20(2.90,5.70) |  | 30629(21250,41747) | 6.41(4.70,8.51) | 52.49(13.41,108.96) |

**Table S5** Incidence, prevalence, and DALYs for multiple sclerosis for all ages in 1990 and 2021, and percentage change in age-standardised rates from 1990 to 2021 for 204 countries and regions

| Nation (or region) | **Incidence** | | | | | **Prevalence** | | | | | **DALYs** | | | | |
| --- | --- | --- | --- | --- | --- | --- | --- | --- | --- | --- | --- | --- | --- | --- | --- |
|  | 1990 counts | 1990 ASR (per 100 000) | 2021 counts | 2021 ASR (per 100 000) | Percentage change in ASR, 1990–2021 | 1990 counts | 1990 ASR (per 100 000) | 2021 counts | 2021 ASR (per 100 000) | Percentage change in ASR, 1990–2021 | 1990 counts | 1990 ASR (per 100 000) | 2021 counts | 2021 ASR (per 100 000) | Percentage change in ASR, 1990–2021 |
| American Samoa | 0(0,0) | 0.17(0.13,0.21) | 0(0,0) | 0.16(0.13,0.20) | 23.41(15.08,31.83) | 1(1,1) | 1.94(1.48,2.49) | 1(1,1) | 2.03(1.56,2.60) | 41.30(30.68,51.47) | 0(0,0) | 0.55(0.36,0.77) | 0(0,0) | 0.57(0.38,0.82) | 69.42(35.80,111.28) |
| Antigua and Barbuda | 0(0,0) | 0.62(0.53,0.72) | 1(1,1) | 0.92(0.82,1.02) | -20.26(-25.04,-14.87) | 7(6,8) | 13.06(10.83,15.41) | 23(20,26) | 20.78(18.21,23.88) | -4.88(-11.11,1.86) | 6(5,7) | 11.90(10.42,13.64) | 17(15,20) | 15.70(13.52,18.03) | -22.21(-49.46,17.00) |
| Arab Republic of Egypt | 347(285,416) | 0.68(0.56,0.81) | 1375(1140,1654) | 1.29(1.07,1.55) | 42.85(31.40,57.19) | 6683(5470,8152) | 16.67(13.64,20.08) | 32170(26475,38915) | 35.97(29.89,43.26) | 53.49(41.98,67.74) | 1943(1333,2676) | 4.81(3.33,6.60) | 8709(5881,12329) | 9.66(6.61,13.62) | 79.29(40.88,121.12) |
| Argentine Republic | 317(271,372) | 0.99(0.85,1.17) | 458(392,540) | 0.96(0.82,1.13) | -3.15(-6.96,0.94) | 7716(6448,9073) | 24.10(20.11,28.35) | 12320(10308,14592) | 24.13(20.13,28.61) | 4.76(0.11,10.04) | 5249(4581,6172) | 16.35(14.27,19.25) | 6378(5248,7623) | 12.44(10.22,14.91) | 4.82(0.21,10.04) |
| Australia | 287(257,321) | 1.57(1.41,1.76) | 601(524,681) | 2.35(2.07,2.67) | 30.07(21.13,39.33) | 7596(6742,8594) | 40.74(36.17,46.10) | 21642(18729,24914) | 64.87(56.31,74.87) | 35.36(25.53,45.85) | 4306(3643,5040) | 23.03(19.50,27.01) | 10236(8455,12323) | 29.72(24.36,36.01) | 8.64(-27.11,59.90) |
| Barbados | 2(1,2) | 0.63(0.53,0.73) | 3(3,3) | 0.92(0.82,1.04) | 2.12(-2.90,7.92) | 33(27,39) | 13.03(10.81,15.36) | 83(72,95) | 20.59(17.78,23.80) | 11.39(6.29,17.62) | 39(35,43) | 15.57(14.09,17.43) | 87(70,107) | 21.68(17.34,26.51) | 32.46(9.96,61.35) |
| Belize | 1(0,1) | 0.37(0.30,0.44) | 2(1,2) | 0.41(0.34,0.49) | 47.65(37.32,62.43) | 8(7,11) | 7.15(5.74,8.82) | 34(27,41) | 8.59(6.98,10.27) | 59.10(47.40,75.78) | 5(4,6) | 3.98(3.30,4.81) | 26(22,30) | 6.44(5.47,7.63) | 31.98(14.73,52.32) |
| Bermuda | 0(0,0) | 0.60(0.49,0.70) | 0(0,1) | 0.68(0.58,0.80) | -3.61(-8.45,1.71) | 9(7,11) | 13.45(11.01,15.99) | 16(13,19) | 16.71(13.86,19.86) | 0.15(-5.37,5.91) | 9(8,10) | 13.01(11.65,14.64) | 11(10,14) | 12.05(10.09,14.46) | -23.90(-31.93,-15.60) |
| Bolivarian Republic of Venezuela | 49(40,60) | 0.30(0.25,0.36) | 108(90,126) | 0.39(0.32,0.45) | 35.56(23.17,52.07) | 737(586,933) | 5.42(4.39,6.70) | 2354(1921,2787) | 7.73(6.32,9.17) | 42.31(30.52,57.85) | 775(695,872) | 5.77(5.18,6.48) | 3577(2832,4507) | 11.85(9.38,14.94) | 17.56(-0.45,37.59) |
| Bosnia and Herzegovina | 60(53,69) | 1.24(1.08,1.40) | 39(35,42) | 1.20(1.10,1.33) | 49.47(36.74,63.32) | 1306(1107,1539) | 27.17(23.13,32.01) | 1267(1151,1401) | 28.21(25.18,31.48) | 59.21(44.50,74.51) | 1266(987,1612) | 26.40(20.68,33.59) | 888(651,1187) | 18.75(13.85,24.99) | 29.02(13.97,46.39) |
| Brunei Darussalam | 1(0,1) | 0.23(0.19,0.28) | 1(1,2) | 0.24(0.20,0.29) | 39.48(28.17,53.18) | 9(7,11) | 4.41(3.54,5.49) | 24(20,30) | 4.82(3.95,5.94) | 43.39(30.69,58.77) | 4(3,7) | 2.10(1.39,3.29) | 11(7,18) | 2.27(1.42,3.66) | 20.83(7.65,34.52) |
| Burkina Faso | 27(22,33) | 0.39(0.32,0.47) | 72(60,87) | 0.40(0.33,0.47) | 3.85(-5.26,12.93) | 390(312,484) | 6.67(5.40,8.22) | 1041(849,1280) | 7.08(5.84,8.58) | 10.67(1.77,19.28) | 318(137,680) | 4.11(2.06,7.98) | 1216(495,2928) | 5.66(2.74,12.33) | -0.81(-19.08,21.17) |
| Canada | 1279(1244,1319) | 4.17(4.06,4.28) | 1661(1622,1710) | 4.74(4.64,4.86) | 33.99(25.35,44.65) | 35002(34122,35959) | 112.21(109.42,115.26) | 68185(66505,69889) | 134.20(130.83,137.70) | 44.57(35.45,55.71) | 16204(13788,18814) | 52.10(44.31,60.48) | 31730(26749,36609) | 59.25(49.05,69.11) | 80.24(50.88,115.63) |
| Central African Republic | 6(5,7) | 0.26(0.21,0.32) | 12(10,15) | 0.26(0.22,0.32) | 2.95(-0.53,7.46) | 76(59,98) | 4.08(3.17,5.10) | 168(132,212) | 4.19(3.31,5.19) | 13.67(9.02,18.18) | 29(20,41) | 1.58(1.12,2.28) | 67(47,96) | 1.75(1.20,2.51) | 22.66(10.05,39.61) |
| Commonwealth of Dominica | 0(0,0) | 0.36(0.30,0.44) | 0(0,0) | 0.40(0.33,0.48) | 47.35(36.05,58.95) | 4(3,5) | 7.35(5.93,9.11) | 7(5,8) | 8.36(6.81,10.02) | 58.08(45.63,71.41) | 3(2,3) | 4.62(3.72,5.71) | 5(4,7) | 6.56(5.00,8.86) | 39.23(11.22,70.18) |
| Commonwealth of the Bahamas | 1(1,2) | 0.52(0.43,0.62) | 3(2,3) | 0.62(0.53,0.73) | -2.95(-9.17,3.74) | 22(18,27) | 10.26(8.31,12.38) | 59(49,71) | 13.29(11.00,15.94) | 21.70(14.00,31.28) | 29(26,32) | 13.78(12.37,15.24) | 89(71,109) | 19.90(15.83,24.38) | -21.28(-35.90,-5.23) |
| Cook Islands | 0(0,0) | 0.19(0.15,0.23) | 0(0,0) | 0.18(0.14,0.21) | 19.81(9.88,28.78) | 0(0,1) | 2.38(1.84,3.03) | 1(0,1) | 2.54(2.01,3.21) | 26.09(14.94,36.68) | 0(0,0) | 0.67(0.44,0.96) | 0(0,0) | 0.72(0.47,1.00) | 16.21(4.17,28.79) |
| Czech Republic | 182(165,200) | 1.70(1.54,1.88) | 140(126,154) | 1.46(1.32,1.63) | 11.28(5.76,16.60) | 4159(3652,4736) | 34.94(30.33,40.01) | 4859(4316,5422) | 35.24(30.88,39.77) | 20.23(13.69,26.81) | 5977(5458,6602) | 48.59(44.31,53.61) | 4264(3625,4995) | 27.97(23.42,33.13) | 61.79(43.82,84.03) |
| Democratic People's Republic of Korea | 54(44,67) | 0.27(0.22,0.33) | 74(61,87) | 0.25(0.20,0.29) | 2.72(-2.59,7.85) | 611(475,786) | 2.96(2.30,3.80) | 1081(852,1348) | 3.41(2.69,4.25) | 8.76(1.43,14.51) | 218(154,304) | 1.07(0.76,1.48) | 404(294,559) | 1.27(0.92,1.77) | 39.57(-36.77,213.67) |
| Democratic Republic of Sao Tome and Principe | 0(0,0) | 0.28(0.23,0.34) | 1(1,1) | 0.31(0.26,0.37) | 14.73(8.06,21.61) | 4(3,5) | 5.04(4.02,6.31) | 10(8,12) | 5.87(4.82,7.09) | 24.21(17.35,31.69) | 2(1,3) | 1.90(1.22,2.89) | 5(3,10) | 2.68(1.73,4.35) | -7.40(-20.57,7.87) |
| Democratic Republic of the Congo | 66(53,83) | 0.23(0.18,0.28) | 173(139,217) | 0.23(0.19,0.28) | 11.52(6.19,17.44) | 940(728,1205) | 3.84(2.96,4.83) | 2555(2003,3307) | 4.08(3.24,5.12) | 19.39(13.54,25.77) | 357(252,509) | 1.50(1.05,2.10) | 1070(756,1468) | 1.78(1.24,2.41) | 28.13(15.92,46.53) |
| Democratic Republic of Timor-Leste | 1(1,1) | 0.17(0.13,0.21) | 2(2,2) | 0.17(0.14,0.21) | 29.71(22.34,37.64) | 11(8,14) | 1.77(1.33,2.28) | 22(16,29) | 1.97(1.51,2.52) | 42.75(32.52,52.59) | 4(2,5) | 0.59(0.41,0.85) | 9(6,12) | 0.78(0.55,1.07) | 105.39(62.31,160.44) |
| Democratic Socialist Republic of Sri Lanka | 28(23,35) | 0.18(0.15,0.22) | 43(36,51) | 0.18(0.15,0.21) | -2.64(-8.05,4.24) | 351(266,465) | 2.24(1.74,2.90) | 621(487,779) | 2.48(1.93,3.13) | 3.81(-3.42,11.84) | 144(108,195) | 0.91(0.69,1.24) | 249(188,329) | 1.00(0.76,1.33) | -28.97(-49.81,5.00) |
| Dominican Republic | 25(20,31) | 0.39(0.32,0.47) | 49(41,59) | 0.43(0.35,0.51) | 2.03(-2.31,7.35) | 383(307,481) | 7.42(6.01,9.22) | 990(803,1183) | 8.99(7.33,10.72) | 8.24(3.80,13.40) | 214(169,271) | 4.06(3.21,5.16) | 587(444,760) | 5.30(4.02,6.85) | 11.31(-3.59,27.23) |
| Eastern Republic of Uruguay | 30(25,35) | 0.98(0.83,1.15) | 36(31,41) | 1.05(0.91,1.22) | 13.07(7.65,19.29) | 795(659,924) | 23.86(19.71,27.87) | 1077(908,1271) | 26.22(22.14,30.92) | 24.54(19.27,30.89) | 714(636,801) | 21.08(18.74,23.67) | 748(641,870) | 17.88(15.28,20.91) | 23.37(16.97,31.39) |
| Federal Democratic Republic of Ethiopia | 105(84,131) | 0.28(0.22,0.34) | 237(190,295) | 0.25(0.21,0.31) | 6.04(1.35,10.81) | 1472(1150,1890) | 4.63(3.64,5.77) | 3526(2763,4449) | 4.77(3.80,5.89) | 9.12(4.32,14.94) | 542(377,781) | 1.74(1.19,2.52) | 1413(949,1940) | 1.96(1.31,2.66) | 7.74(-14.12,44.21) |
| Federal Democratic Republic of Nepal | 66(53,82) | 0.40(0.33,0.49) | 140(118,167) | 0.45(0.37,0.53) | -1.76(-6.52,3.61) | 1033(832,1307) | 7.43(5.96,9.24) | 2543(2082,3049) | 8.98(7.38,10.72) | 8.88(2.59,15.37) | 326(229,457) | 2.36(1.66,3.27) | 858(612,1184) | 3.04(2.16,4.21) | -7.86(-21.96,8.32) |
| Federal Republic of Germany | 2294(2016,2597) | 2.73(2.43,3.09) | 2524(2241,2860) | 3.29(2.94,3.76) | 2.33(-3.77,7.68) | 72452(62928,84885) | 71.64(62.26,84.00) | 107807(94780,126152) | 90.46(79.32,105.13) | 6.15(1.12,12.33) | 45575(39894,52434) | 43.82(38.04,50.96) | 61999(53738,71359) | 47.18(40.28,54.85) | 37.69(-37.89,217.87) |
| Federal Republic of Nigeria | 234(190,289) | 0.32(0.26,0.39) | 710(587,853) | 0.38(0.31,0.45) | -3.71(-8.19,-0.12) | 3483(2758,4343) | 5.66(4.52,6.96) | 10901(8940,13310) | 7.25(5.94,8.73) | 0.02(-4.39,4.93) | 3062(1921,4473) | 3.62(2.43,5.17) | 13481(8445,21676) | 5.90(3.90,8.96) | 5.34(-8.23,25.63) |
| Federal Republic of Somalia | 15(12,19) | 0.25(0.20,0.31) | 40(32,51) | 0.25(0.20,0.30) | -2.73(-7.36,1.72) | 203(155,268) | 4.08(3.18,5.24) | 538(420,696) | 4.14(3.29,5.15) | 11.95(6.20,17.37) | 69(47,101) | 1.42(0.97,2.05) | 189(127,268) | 1.50(0.99,2.13) | 34.10(15.60,64.03) |
| Federated States of Micronesia | 0(0,0) | 0.18(0.14,0.21) | 0(0,0) | 0.17(0.14,0.21) | 8.27(3.01,14.56) | 1(1,2) | 1.62(1.22,2.11) | 2(1,2) | 1.78(1.35,2.28) | 13.73(8.02,21.10) | 0(0,1) | 0.46(0.30,0.66) | 0(0,1) | 0.50(0.33,0.72) | 40.87(-46.53,275.52) |
| Federative Republic of Brazil | 1071(901,1266) | 0.80(0.67,0.93) | 2254(1910,2601) | 0.91(0.77,1.05) | 13.66(10.78,16.13) | 19070(15878,22730) | 17.16(14.42,20.29) | 54710(46538,64297) | 21.38(18.22,25.15) | 19.60(16.78,22.32) | 8625(7038,10606) | 7.78(6.34,9.56) | 24513(20052,29656) | 9.60(7.85,11.61) | 13.72(4.85,23.19) |
| French Republic | 1329(1149,1553) | 2.25(1.95,2.64) | 1687(1513,1858) | 3.05(2.72,3.37) | 0.27(-3.91,4.55) | 42142(36018,49498) | 62.81(53.60,73.99) | 78617(69623,87964) | 91.81(81.52,103.14) | 2.79(-2.43,7.81) | 22658(19248,26559) | 33.42(28.25,39.30) | 36595(30647,43408) | 40.36(33.20,47.82) | 10.61(-3.91,31.44) |
| Gabonese Republic | 2(1,2) | 0.23(0.18,0.28) | 4(3,5) | 0.24(0.20,0.29) | 4.07(-1.09,9.41) | 27(21,34) | 3.88(3.06,4.83) | 66(52,83) | 4.40(3.51,5.43) | 5.88(0.02,12.22) | 12(9,15) | 1.70(1.27,2.20) | 32(24,43) | 2.22(1.64,2.94) | 38.45(-35.30,188.48) |
| Georgia | 51(41,60) | 0.89(0.72,1.05) | 43(37,49) | 1.10(0.96,1.25) | 2.30(-2.85,7.19) | 1171(956,1387) | 18.96(15.52,22.47) | 1225(1059,1397) | 24.29(21.02,27.86) | 7.42(2.45,12.72) | 432(331,556) | 7.17(5.54,9.13) | 445(338,564) | 8.67(6.55,10.99) | -22.18(-31.18,-12.04) |
| Grand Duchy of Luxembourg | 12(11,14) | 2.90(2.58,3.29) | 22(19,25) | 3.31(2.91,3.74) | -8.44(-12.39,-4.11) | 353(305,411) | 74.87(64.78,87.52) | 777(673,893) | 90.15(78.27,103.78) | 43.29(37.03,50.23) | 214(182,248) | 44.36(37.67,51.66) | 382(323,447) | 42.58(35.60,50.09) | 33.57(11.50,54.02) |
| Greenland | 1(1,1) | 1.66(1.44,1.92) | 1(1,1) | 1.73(1.50,1.98) | 25.36(18.87,32.75) | 35(30,40) | 56.28(48.35,65.02) | 39(33,44) | 60.25(51.76,69.42) | 43.85(35.66,53.14) | 10(7,13) | 16.51(11.87,21.52) | 14(11,19) | 21.49(16.61,27.94) | 43.65(21.95,66.01) |
| Grenada | 0(0,0) | 0.52(0.44,0.61) | 1(1,1) | 0.64(0.55,0.74) | 20.36(13.42,28.23) | 6(5,8) | 10.03(8.16,11.94) | 15(13,18) | 13.11(10.94,15.50) | 29.57(22.17,38.80) | 9(8,10) | 13.65(12.18,15.32) | 19(16,22) | 16.08(13.54,18.73) | 44.37(13.03,79.29) |
| Guam | 0(0,0) | 0.15(0.12,0.19) | 0(0,0) | 0.15(0.12,0.18) | 0.67(-4.11,5.89) | 2(2,3) | 1.97(1.52,2.52) | 4(3,5) | 2.04(1.57,2.60) | 6.05(0.85,11.56) | 1(0,1) | 0.56(0.37,0.80) | 1(1,1) | 0.57(0.38,0.81) | 24.38(4.33,53.29) |
| Hashemite Kingdom of Jordan | 59(51,67) | 1.69(1.47,1.93) | 252(210,301) | 1.86(1.55,2.22) | 2.22(-2.35,7.61) | 1051(912,1195) | 48.39(42.36,54.99) | 5970(4882,7224) | 54.67(44.83,65.73) | 8.99(3.29,14.73) | 462(359,588) | 20.94(16.51,26.77) | 2411(1870,3052) | 21.74(16.96,27.43) | 25.62(1.99,58.34) |
| Hellenic Republic | 108(95,122) | 1.03(0.90,1.16) | 130(114,145) | 1.46(1.28,1.67) | -7.15(-10.22,-3.22) | 3035(2629,3443) | 24.59(21.31,28.10) | 5250(4498,6132) | 37.62(32.25,44.22) | 6.70(1.40,11.91) | 2292(2010,2607) | 17.75(15.48,20.30) | 4484(3942,5037) | 29.17(25.51,33.07) | 6.77(1.41,11.89) |
| Hungary | 205(187,222) | 1.94(1.78,2.10) | 123(111,137) | 1.43(1.30,1.61) | 34.56(27.03,45.42) | 5576(5112,6069) | 44.69(40.78,48.61) | 4417(3870,5018) | 35.52(30.91,40.58) | 47.61(37.49,59.82) | 5425(4872,6026) | 42.82(38.41,47.79) | 3699(3175,4234) | 27.03(23.08,31.22) | 87.66(65.31,111.91) |
| Independent State of Papua New Guinea | 5(4,6) | 0.15(0.12,0.19) | 13(10,16) | 0.15(0.11,0.18) | 35.42(28.69,44.49) | 45(33,62) | 1.41(1.05,1.85) | 133(97,180) | 1.45(1.08,1.91) | 56.11(46.90,68.66) | 13(8,19) | 0.40(0.25,0.58) | 38(24,56) | 0.41(0.27,0.60) | -25.11(-36.40,-13.01) |
| Independent State of Samoa | 0(0,0) | 0.18(0.14,0.22) | 0(0,0) | 0.17(0.14,0.21) | 25.99(17.60,33.95) | 2(2,3) | 1.97(1.51,2.53) | 4(3,5) | 2.09(1.61,2.65) | 36.60(26.92,45.87) | 1(0,1) | 0.56(0.37,0.79) | 1(1,2) | 0.59(0.39,0.84) | 25.48(9.27,43.49) |
| Ireland | 137(123,154) | 3.89(3.46,4.36) | 196(173,227) | 4.44(3.93,5.20) | 41.75(31.58,54.52) | 3995(3522,4577) | 110.10(97.10,126.17) | 8036(6928,9630) | 129.81(111.46,155.56) | 52.09(38.80,67.10) | 2130(1801,2454) | 58.22(49.14,67.01) | 3782(3102,4471) | 58.83(47.78,69.95) | 20.16(-16.00,76.03) |
| Islamic Republic of Afghanistan | 105(89,124) | 1.42(1.22,1.66) | 503(423,595) | 1.76(1.51,2.04) | -13.95(-17.40,-10.17) | 2334(1948,2742) | 33.32(27.46,39.39) | 8654(7243,10270) | 47.08(40.05,55.38) | 0.87(-4.79,7.88) | 828(564,1166) | 11.64(7.88,16.64) | 3720(2622,5164) | 19.72(13.90,27.03) | -42.45(-50.87,-33.16) |
| Islamic Republic of Iran | 1032(872,1208) | 2.08(1.79,2.41) | 2070(1799,2356) | 2.09(1.84,2.35) | -8.52(-13.48,-4.05) | 19404(16223,22955) | 53.59(45.23,62.60) | 52793(45840,60642) | 55.31(48.26,63.17) | 14.92(7.79,20.98) | 8500(6147,11030) | 22.91(16.69,29.66) | 26879(22408,31806) | 27.97(23.30,33.11) | 18.99(3.64,39.65) |
| Islamic Republic of Mauritania | 8(6,9) | 0.48(0.40,0.57) | 18(15,22) | 0.49(0.42,0.57) | 1.24(-3.01,5.46) | 116(94,142) | 8.66(6.98,10.38) | 291(240,351) | 9.57(7.99,11.36) | 6.21(0.36,11.74) | 107(56,209) | 5.68(3.36,10.18) | 358(162,762) | 8.18(4.16,16.23) | 18.74(0.95,43.59) |
| Islamic Republic of Pakistan | 401(329,486) | 0.45(0.37,0.54) | 1127(937,1342) | 0.50(0.42,0.59) | 7.31(-0.32,15.92) | 6269(5093,7704) | 8.34(6.76,10.17) | 18628(15203,22471) | 9.80(8.12,11.62) | 13.47(4.18,24.68) | 2043(1460,2831) | 2.73(1.95,3.76) | 6512(4799,8636) | 3.46(2.54,4.57) | -5.73(-16.83,5.30) |
| Jamaica | 8(7,10) | 0.38(0.31,0.46) | 13(11,15) | 0.41(0.34,0.50) | 3.22(-2.04,8.29) | 142(114,174) | 7.78(6.24,9.60) | 279(229,337) | 9.04(7.42,10.93) | 7.87(2.39,12.97) | 90(76,108) | 5.03(4.27,5.96) | 250(197,318) | 8.12(6.39,10.31) | 23.46(2.48,53.72) |
| Japan | 501(407,600) | 0.37(0.30,0.44) | 500(414,592) | 0.39(0.32,0.47) | 9.39(3.29,14.26) | 13549(11045,16553) | 8.64(7.01,10.58) | 18475(15245,22042) | 9.42(7.71,11.42) | 13.70(6.39,19.27) | 5662(4396,7227) | 3.61(2.79,4.62) | 7500(5856,9608) | 3.85(3.01,4.92) | 41.92(11.05,81.54) |
| Kingdom of Bahrain | 6(5,7) | 0.96(0.79,1.16) | 25(20,30) | 1.29(1.06,1.55) | 10.92(3.36,17.82) | 102(81,126) | 25.78(20.95,31.18) | 622(504,759) | 37.27(30.62,45.30) | 21.16(13.25,29.54) | 28(19,39) | 7.05(4.74,9.72) | 213(159,280) | 12.70(9.59,16.51) | 30.67(3.05,78.31) |
| Kingdom of Belgium | 261(233,289) | 2.52(2.26,2.79) | 316(274,362) | 3.01(2.63,3.46) | 33.17(26.55,41.59) | 8060(7156,9062) | 66.04(58.64,74.36) | 12739(10994,14856) | 83.27(72.06,97.55) | 47.54(39.44,57.51) | 4910(4286,5575) | 38.94(33.68,44.41) | 7314(6241,8483) | 45.25(37.71,52.90) | 55.78(31.88,88.41) |
| Kingdom of Bhutan | 2(2,3) | 0.39(0.32,0.47) | 4(3,4) | 0.44(0.37,0.51) | 90.59(76.92,106.31) | 30(25,38) | 7.42(6.00,8.98) | 67(55,81) | 8.86(7.32,10.56) | 115.77(100.89,133.42) | 10(7,14) | 2.37(1.66,3.30) | 23(16,31) | 3.03(2.18,4.13) | 100.56(71.53,133.23) |
| Kingdom of Cambodia | 15(12,19) | 0.20(0.16,0.24) | 32(26,39) | 0.20(0.16,0.23) | 17.83(12.14,24.73) | 147(110,196) | 1.99(1.54,2.59) | 369(281,475) | 2.23(1.71,2.82) | 36.09(28.74,44.76) | 53(38,76) | 0.72(0.51,1.02) | 157(111,222) | 0.96(0.67,1.34) | 83.85(48.20,137.07) |
| Kingdom of Denmark | 210(193,226) | 3.95(3.64,4.29) | 222(198,250) | 4.24(3.81,4.79) | 1.46(-3.94,6.90) | 6393(5784,7029) | 101.78(91.52,111.89) | 8751(7713,10177) | 115.48(101.37,134.72) | 17.05(9.62,24.59) | 4518(4027,5142) | 69.78(62.15,79.44) | 5525(4780,6334) | 65.78(56.60,76.05) | 51.20(8.65,121.49) |
| Kingdom of Eswatini | 2(2,3) | 0.36(0.29,0.43) | 4(3,5) | 0.37(0.31,0.43) | 2.16(-2.78,6.96) | 31(24,39) | 6.41(5.09,7.87) | 62(51,77) | 6.85(5.55,8.28) | 8.27(2.51,13.99) | 10(7,14) | 2.17(1.55,2.94) | 22(16,30) | 2.56(1.89,3.39) | 27.55(8.39,57.58) |
| Kingdom of Lesotho | 5(4,6) | 0.37(0.30,0.45) | 7(6,8) | 0.39(0.33,0.46) | -14.96(-19.43,-11.15) | 72(57,90) | 6.71(5.31,8.33) | 109(89,133) | 7.15(5.85,8.57) | 6.32(-0.49,13.01) | 23(16,32) | 2.17(1.55,3.04) | 38(28,52) | 2.57(1.90,3.46) | -59.42(-66.60,-52.44) |
| Kingdom of Morocco | 299(242,358) | 1.22(1.01,1.45) | 694(587,819) | 1.78(1.50,2.10) | -8.23(-10.70,-5.63) | 5879(4738,7060) | 31.70(25.79,38.00) | 19170(15994,23019) | 49.81(41.55,59.84) | 2.87(-0.16,6.41) | 2005(1435,2745) | 10.70(7.52,14.65) | 7888(5861,10712) | 20.38(15.15,27.49) | 12.83(-4.13,35.83) |
| Kingdom of Norway | 147(129,168) | 3.34(2.92,3.84) | 258(222,295) | 4.87(4.18,5.61) | -2.82(-6.09,1.70) | 4152(3541,4830) | 82.81(70.69,96.87) | 9510(8099,11153) | 131.53(111.04,154.17) | 9.77(4.26,15.05) | 2874(2544,3239) | 55.70(49.10,62.97) | 4806(4030,5614) | 62.94(52.32,74.19) | 9.83(4.38,15.65) |
| Kingdom of Saudi Arabia | 134(107,163) | 0.86(0.71,1.04) | 584(466,718) | 1.17(0.97,1.43) | -3.45(-7.68,0.72) | 2325(1858,2872) | 22.11(17.97,26.80) | 13365(10761,16516) | 32.85(27.05,40.43) | 4.19(-0.59,9.11) | 730(516,1005) | 6.86(4.83,9.28) | 4770(3529,6393) | 11.25(8.49,14.92) | 4.23(-0.47,9.53) |
| Kingdom of Spain | 714(641,798) | 1.80(1.61,2.02) | 974(886,1065) | 2.49(2.27,2.70) | 20.08(13.45,28.48) | 22066(19654,24667) | 49.71(44.20,55.71) | 47876(43605,51956) | 74.54(67.82,81.17) | 24.93(17.46,33.69) | 10018(8336,11977) | 22.35(18.45,26.72) | 19025(15338,22910) | 28.51(22.75,34.42) | 9.30(-0.99,19.99) |
| Kingdom of Sweden | 372(326,425) | 4.41(3.89,5.04) | 537(471,605) | 5.58(4.91,6.34) | 35.25(24.92,47.58) | 13768(11998,15848) | 125.99(110.11,144.47) | 22713(19839,26016) | 161.60(140.22,186.95) | 46.18(33.63,61.43) | 5935(4934,7124) | 53.60(44.08,64.83) | 9274(7563,11245) | 63.51(51.03,77.77) | 20.77(10.26,32.64) |
| Kingdom of Thailand | 101(81,126) | 0.19(0.15,0.23) | 148(119,178) | 0.18(0.15,0.22) | 6.54(0.95,12.04) | 1260(960,1652) | 2.38(1.84,3.02) | 2295(1815,2850) | 2.60(2.04,3.29) | 13.34(7.48,19.28) | 435(302,606) | 0.82(0.57,1.13) | 888(661,1179) | 1.04(0.77,1.36) | 30.33(0.02,80.61) |
| Kingdom of the Netherlands | 499(438,560) | 3.05(2.69,3.42) | 537(479,609) | 3.48(3.11,3.94) | 24.30(11.16,39.23) | 14157(12222,16405) | 80.35(69.42,93.19) | 22160(19394,25758) | 95.89(83.35,111.16) | 28.08(15.99,42.23) | 8404(7285,9638) | 47.08(40.70,53.97) | 12709(10900,14560) | 50.87(42.85,58.81) | 20.95(5.65,39.14) |
| Kingdom of Tonga | 0(0,0) | 0.19(0.15,0.23) | 0(0,0) | 0.18(0.15,0.22) | 20.27(11.41,29.66) | 2(1,2) | 2.29(1.76,2.95) | 2(2,3) | 2.43(1.89,3.07) | 26.27(14.81,36.96) | 0(0,1) | 0.65(0.42,0.93) | 1(0,1) | 0.69(0.46,0.98) | 7.67(-2.07,18.29) |
| Kyrgyz Republic | 32(26,37) | 0.87(0.71,1.03) | 56(47,66) | 0.85(0.70,0.99) | 63.40(49.79,78.35) | 577(480,684) | 17.36(14.33,20.53) | 1077(908,1262) | 18.28(15.43,21.37) | 80.14(62.70,101.36) | 254(202,312) | 7.75(6.18,9.50) | 412(319,526) | 7.24(5.61,9.24) | 54.33(-27.20,237.18) |
| Lao People's Democratic Republic | 7(6,9) | 0.23(0.18,0.27) | 14(12,18) | 0.21(0.17,0.26) | 42.00(33.59,52.30) | 68(51,90) | 2.19(1.68,2.87) | 168(128,221) | 2.38(1.85,3.09) | 53.00(42.91,65.75) | 24(17,35) | 0.77(0.54,1.12) | 70(48,98) | 1.00(0.69,1.40) | 64.33(48.29,81.88) |
| Lebanese Republic | 41(34,49) | 1.43(1.18,1.71) | 131(110,159) | 2.07(1.75,2.49) | 3.66(-5.28,13.57) | 970(801,1177) | 38.63(32.02,46.73) | 3743(3113,4574) | 61.41(51.19,74.92) | 7.05(-2.51,17.73) | 336(249,458) | 13.30(9.83,18.12) | 1193(926,1566) | 19.60(15.15,25.71) | 30.13(9.10,58.32) |
| Malaysia | 22(17,28) | 0.15(0.12,0.19) | 50(40,61) | 0.15(0.12,0.18) | 21.49(15.02,29.00) | 261(193,343) | 1.79(1.37,2.30) | 673(522,865) | 1.99(1.56,2.52) | 30.71(23.04,39.50) | 134(91,183) | 0.94(0.63,1.30) | 429(331,546) | 1.28(0.99,1.63) | 17.80(-0.70,39.21) |
| Mongolia | 16(13,19) | 0.95(0.77,1.13) | 30(25,36) | 0.87(0.73,1.03) | -6.03(-9.77,-2.13) | 232(192,279) | 17.46(14.33,21.05) | 556(464,663) | 18.30(15.28,21.65) | 3.24(-1.24,8.38) | 131(83,201) | 10.65(6.69,16.87) | 329(215,504) | 11.94(7.82,18.51) | 3.23(-1.24,8.45) |
| Montenegro | 12(11,14) | 1.90(1.73,2.10) | 13(12,14) | 2.15(1.98,2.32) | 28.94(20.85,37.35) | 272(239,309) | 41.54(36.43,47.24) | 405(369,445) | 50.94(45.71,56.65) | 49.81(40.43,59.84) | 229(167,309) | 34.86(25.48,47.33) | 289(226,359) | 35.33(27.40,44.01) | 55.56(37.23,75.11) |
| New Zealand | 57(49,66) | 1.57(1.35,1.81) | 73(65,83) | 1.40(1.24,1.59) | 6.52(1.80,12.95) | 1393(1163,1621) | 38.13(31.82,44.34) | 2219(1910,2555) | 34.71(29.84,40.32) | 11.02(5.63,17.65) | 963(835,1108) | 26.22(22.72,30.14) | 1647(1406,1879) | 24.09(20.43,27.58) | 62.21(-26.53,250.36) |
| North Macedonia | 29(26,33) | 1.39(1.24,1.58) | 42(38,46) | 1.79(1.62,1.99) | 6.71(0.73,14.20) | 636(545,740) | 30.84(26.48,35.81) | 1315(1165,1474) | 44.41(38.94,50.13) | 13.13(6.76,21.16) | 581(455,734) | 28.15(21.93,35.65) | 826(644,1059) | 27.22(21.22,34.76) | 44.01(-38.95,216.07) |
| Northern Mariana Islands | 0(0,0) | 0.18(0.14,0.21) | 0(0,0) | 0.17(0.14,0.21) | 9.41(4.08,15.65) | 1(1,1) | 2.17(1.67,2.75) | 1(1,2) | 2.33(1.82,2.94) | 22.01(15.08,30.26) | 0(0,0) | 0.61(0.40,0.86) | 0(0,1) | 0.66(0.43,0.93) | 54.95(19.11,97.42) |
| Palestine | 22(18,26) | 1.26(1.06,1.51) | 92(76,109) | 1.72(1.44,2.04) | 7.48(2.05,13.83) | 403(328,485) | 34.60(28.31,41.65) | 1909(1561,2284) | 49.61(40.93,59.23) | 19.36(12.65,28.54) | 161(119,222) | 13.77(10.09,18.75) | 880(690,1097) | 22.91(18.21,28.62) | 33.54(-11.48,97.02) |
| People's Democratic Republic of Algeria | 291(239,349) | 1.26(1.05,1.50) | 836(717,982) | 1.81(1.55,2.12) | 21.44(15.19,28.56) | 5472(4483,6580) | 33.28(27.63,39.99) | 22398(18925,26657) | 51.08(43.25,60.58) | 34.12(27.25,43.13) | 1899(1403,2580) | 11.41(8.44,15.56) | 9031(6982,11602) | 20.45(16.05,26.18) | 34.34(8.48,67.22) |
| People's Republic of Bangladesh | 331(268,407) | 0.37(0.30,0.45) | 653(539,785) | 0.38(0.32,0.46) | -26.06(-30.69,-21.19) | 4881(3909,6094) | 6.87(5.50,8.40) | 12571(10256,15420) | 7.81(6.38,9.49) | -20.52(-26.74,-13.65) | 1580(1116,2190) | 2.25(1.59,3.09) | 4417(3163,6019) | 2.75(1.97,3.74) | -36.88(-44.48,-26.67) |
| People's Republic of China | 1961(1546,2436) | 0.18(0.14,0.22) | 2795(2250,3347) | 0.16(0.13,0.20) | 3.49(-6.33,17.27) | 17887(13402,23693) | 1.55(1.19,2.01) | 40159(31807,50038) | 2.23(1.74,2.83) | 9.39(-2.96,25.61) | 7016(5078,9796) | 0.62(0.46,0.87) | 15005(11002,19590) | 0.83(0.61,1.09) | 10.52(-2.89,23.42) |
| Plurinational State of Bolivia | 21(17,26) | 0.41(0.34,0.49) | 66(55,78) | 0.56(0.47,0.65) | 10.99(6.20,15.19) | 331(265,410) | 7.66(6.20,9.32) | 1242(1037,1476) | 11.50(9.63,13.55) | 19.13(14.22,23.58) | 200(135,287) | 4.73(3.16,6.85) | 797(564,1128) | 7.48(5.30,10.47) | 28.82(17.43,40.69) |
| Portuguese Republic | 126(108,146) | 1.22(1.04,1.42) | 120(109,132) | 1.20(1.10,1.31) | 1.04(-2.99,5.76) | 3579(3004,4212) | 30.73(25.73,36.40) | 4872(4460,5356) | 31.62(28.76,34.69) | 9.23(5.69,12.41) | 2194(1880,2558) | 18.39(15.64,21.55) | 2966(2554,3406) | 18.37(15.77,21.32) | 32.36(15.00,60.26) |
| Principality of Andorra | 2(1,2) | 2.38(2.10,2.66) | 3(2,3) | 3.09(2.73,3.51) | 31.01(21.97,39.92) | 39(34,45) | 61.83(53.71,71.07) | 106(92,122) | 83.70(72.91,97.08) | 40.84(31.55,51.16) | 23(17,32) | 37.35(27.80,52.17) | 54(39,72) | 40.58(29.39,54.65) | 38.76(14.98,69.03) |
| Principality of Monaco | 0(0,1) | 1.65(1.43,1.93) | 1(1,1) | 2.00(1.73,2.29) | 14.12(4.29,24.66) | 20(17,23) | 45.25(38.68,53.83) | 31(27,36) | 56.59(48.23,65.62) | 17.90(6.23,30.79) | 8(6,11) | 18.14(13.67,23.54) | 14(10,20) | 24.86(18.35,33.04) | 1.05(-10.37,12.19) |
| Puerto Rico | 18(14,21) | 0.48(0.39,0.57) | 21(18,24) | 0.62(0.53,0.74) | 0.48(-3.70,5.29) | 364(296,434) | 10.13(8.25,12.09) | 640(535,762) | 14.19(11.80,16.85) | 3.20(-0.64,7.66) | 471(432,521) | 13.11(12.04,14.52) | 638(527,763) | 14.58(12.05,17.44) | 22.06(-0.08,54.65) |
| Republic of Albania | 125(115,136) | 4.45(4.12,4.83) | 98(91,105) | 3.55(3.28,3.82) | 15.03(8.04,21.13) | 2165(1958,2389) | 88.63(80.72,97.43) | 3086(2831,3379) | 84.30(76.46,93.33) | 20.98(13.86,27.65) | 1875(1422,2412) | 72.84(55.52,93.00) | 2008(1426,2924) | 56.66(40.30,82.29) | 12.12(1.30,25.20) |
| Republic of Angola | 23(18,29) | 0.28(0.23,0.34) | 74(60,92) | 0.29(0.23,0.34) | 24.39(19.80,29.33) | 318(248,411) | 4.67(3.67,5.88) | 1093(876,1393) | 5.20(4.21,6.46) | 32.48(27.04,38.30) | 118(82,168) | 1.79(1.23,2.57) | 479(335,677) | 2.37(1.63,3.33) | 24.25(17.37,30.46) |
| Republic of Armenia | 35(29,40) | 1.08(0.91,1.25) | 48(43,54) | 1.47(1.30,1.65) | 8.46(2.50,14.23) | 697(596,801) | 22.71(19.37,26.16) | 1294(1138,1461) | 32.32(28.28,36.59) | 16.21(9.75,23.19) | 292(228,368) | 9.86(7.73,12.33) | 462(361,580) | 11.59(9.04,14.39) | 61.59(31.10,98.72) |
| Republic of Austria | 178(154,205) | 2.17(1.89,2.51) | 258(229,292) | 3.03(2.70,3.39) | 4.75(1.66,7.75) | 5471(4607,6426) | 58.13(48.93,68.72) | 10323(9116,11788) | 83.35(73.13,95.09) | 8.99(5.81,12.50) | 3445(2992,4009) | 35.91(31.01,42.09) | 5743(4833,6636) | 43.38(36.01,50.62) | 6.66(3.68,9.61) |
| Republic of Azerbaijan | 54(45,64) | 0.84(0.68,0.99) | 105(87,123) | 0.87(0.73,1.02) | 10.43(1.76,21.47) | 1000(827,1191) | 17.07(14.09,20.20) | 2257(1912,2653) | 18.89(16.04,22.18) | 12.98(2.40,23.90) | 389(295,512) | 6.79(5.21,8.90) | 777(574,1034) | 6.73(5.09,8.87) | 3.84(-17.48,33.36) |
| Republic of Belarus | 86(73,100) | 0.79(0.68,0.92) | 73(64,83) | 0.77(0.68,0.88) | 7.61(-1.03,17.67) | 1575(1331,1881) | 13.27(11.15,15.85) | 2020(1763,2325) | 16.15(14.04,18.79) | 13.43(4.52,23.69) | 2432(2196,2697) | 21.00(18.98,23.30) | 2061(1657,2488) | 16.54(13.32,19.98) | -19.77(-35.15,-5.48) |
| Republic of Benin | 13(10,16) | 0.37(0.30,0.45) | 41(34,50) | 0.38(0.32,0.45) | 10.72(6.34,15.42) | 183(145,229) | 6.39(5.15,7.86) | 600(494,735) | 6.95(5.75,8.36) | 13.56(9.43,18.09) | 165(77,345) | 4.09(2.22,7.83) | 762(348,1646) | 5.70(3.04,11.36) | 34.00(17.75,57.12) |
| Republic of Botswana | 3(3,4) | 0.33(0.27,0.40) | 8(7,10) | 0.34(0.28,0.40) | 3.06(-2.80,8.88) | 50(39,63) | 5.90(4.70,7.23) | 144(117,181) | 6.39(5.18,7.81) | 6.93(1.50,13.55) | 17(12,23) | 2.01(1.44,2.73) | 50(36,65) | 2.24(1.62,2.96) | 18.06(0.92,41.78) |
| Republic of Bulgaria | 214(196,231) | 2.27(2.07,2.45) | 157(145,169) | 2.23(2.05,2.41) | 0.18(-4.25,4.94) | 5287(4759,5858) | 46.40(41.34,52.11) | 5137(4682,5633) | 50.52(45.56,56.03) | 11.05(5.29,17.59) | 4204(3754,4712) | 38.59(34.48,43.23) | 3531(3009,4127) | 35.55(30.33,41.68) | 11.19(5.54,17.85) |
| Republic of Burundi | 10(8,13) | 0.24(0.20,0.30) | 25(20,32) | 0.23(0.19,0.28) | 64.71(54.47,76.90) | 140(109,178) | 3.97(3.10,4.90) | 353(272,455) | 3.97(3.12,4.93) | 77.21(65.53,91.56) | 52(36,73) | 1.50(1.03,2.12) | 136(92,187) | 1.58(1.05,2.18) | 94.86(66.76,132.19) |
| Republic of Cabo Verde | 1(1,1) | 0.38(0.31,0.46) | 3(2,3) | 0.42(0.35,0.49) | -2.38(-9.58,3.70) | 17(14,21) | 7.67(6.30,9.42) | 48(40,58) | 8.67(7.25,10.35) | 5.34(-1.65,12.06) | 19(7,41) | 5.46(2.59,10.50) | 37(18,77) | 6.10(3.11,12.36) | -6.52(-18.51,7.01) |
| Republic of Cameroon | 28(23,34) | 0.36(0.29,0.43) | 103(85,124) | 0.39(0.32,0.45) | -6.57(-10.66,-3.14) | 395(318,494) | 5.95(4.77,7.31) | 1465(1183,1803) | 6.76(5.49,8.11) | 8.82(3.58,14.09) | 452(226,873) | 4.68(2.58,8.48) | 2155(909,4935) | 6.60(3.12,14.17) | 30.20(11.95,60.43) |
| Republic of Chad | 18(15,22) | 0.41(0.33,0.49) | 54(45,65) | 0.42(0.36,0.50) | -5.80(-9.81,-1.00) | 256(203,318) | 6.91(5.62,8.54) | 730(591,910) | 7.32(6.02,9.03) | 15.57(8.44,23.18) | 174(82,372) | 3.59(1.97,6.85) | 763(317,1714) | 4.97(2.51,9.83) | -47.21(-56.05,-38.11) |
| Republic of Chile | 103(83,123) | 0.73(0.60,0.87) | 149(123,179) | 0.75(0.62,0.90) | 44.48(32.51,56.63) | 2235(1802,2691) | 18.65(15.04,22.44) | 4557(3716,5510) | 20.04(16.29,24.24) | 58.98(45.53,72.58) | 1222(1014,1466) | 10.38(8.65,12.44) | 1849(1456,2320) | 8.08(6.36,10.18) | 47.32(19.09,79.13) |
| Republic of Colombia | 73(57,90) | 0.25(0.20,0.30) | 168(139,201) | 0.31(0.26,0.37) | 6.79(-0.59,14.67) | 1137(882,1434) | 4.65(3.68,5.78) | 3643(2985,4391) | 6.69(5.47,8.07) | 6.50(0.14,14.15) | 1176(1052,1317) | 4.76(4.29,5.34) | 3723(3120,4420) | 6.84(5.73,8.11) | 18.69(4.00,38.75) |
| Republic of Costa Rica | 10(8,12) | 0.36(0.30,0.43) | 25(22,30) | 0.48(0.41,0.56) | 3.14(-1.68,8.73) | 160(129,199) | 7.24(5.86,8.78) | 581(481,693) | 10.69(8.86,12.74) | 12.06(6.51,18.24) | 127(111,148) | 5.77(5.07,6.66) | 586(508,671) | 10.82(9.39,12.40) | 64.32(-32.44,257.22) |
| Republic of Croatia | 53(46,59) | 1.01(0.90,1.14) | 54(49,59) | 1.37(1.25,1.52) | 49.71(38.78,61.90) | 1218(1046,1408) | 20.81(17.85,24.17) | 1833(1647,2059) | 32.48(28.61,36.90) | 60.03(49.40,72.61) | 1863(1676,2068) | 31.13(28.09,34.58) | 1436(1220,1650) | 23.31(19.70,27.12) | 147.42(86.20,237.13) |
| Republic of Cuba | 61(51,72) | 0.52(0.44,0.62) | 77(66,88) | 0.66(0.56,0.76) | -6.51(-10.73,-1.94) | 1194(979,1420) | 11.11(9.14,13.18) | 2338(1958,2733) | 15.18(12.65,17.89) | 14.37(7.46,22.17) | 1336(1217,1489) | 12.35(11.23,13.74) | 2449(2096,2853) | 15.49(13.26,18.02) | -43.65(-53.18,-33.01) |
| Republic of Cyprus | 10(9,11) | 1.25(1.11,1.40) | 26(22,31) | 1.77(1.52,2.04) | 14.17(5.80,23.66) | 264(236,301) | 32.33(28.84,36.84) | 883(747,1039) | 49.18(41.43,57.96) | 20.40(11.39,32.27) | 171(111,245) | 20.99(13.65,30.26) | 463(361,592) | 25.22(19.65,32.18) | -4.02(-14.59,7.23) |
| Republic of Cmete d'Ivoire | 33(26,41) | 0.35(0.29,0.42) | 87(72,105) | 0.36(0.30,0.42) | -1.95(-6.30,2.32) | 432(343,544) | 5.73(4.59,7.03) | 1265(1028,1549) | 6.31(5.15,7.64) | 2.88(-1.84,7.97) | 454(206,997) | 3.92(2.11,7.87) | 1655(728,3920) | 5.90(2.91,13.12) | 12.68(-2.64,36.66) |
| Republic of Djibouti | 1(1,1) | 0.27(0.22,0.33) | 4(3,4) | 0.28(0.23,0.34) | -0.67(-4.53,3.32) | 13(10,17) | 4.86(3.85,6.03) | 59(48,74) | 5.25(4.25,6.41) | 6.22(2.13,10.99) | 5(3,7) | 1.87(1.24,2.70) | 25(17,36) | 2.30(1.53,3.30) | 25.13(9.11,50.27) |
| Republic of Ecuador | 22(18,28) | 0.27(0.22,0.32) | 65(54,78) | 0.36(0.30,0.43) | -1.43(-5.82,3.01) | 354(278,448) | 5.07(3.97,6.29) | 1306(1061,1604) | 7.48(6.09,9.13) | 11.10(6.26,17.46) | 272(237,316) | 3.96(3.47,4.58) | 1069(870,1298) | 6.17(5.03,7.50) | 36.30(0.79,86.86) |
| Republic of El Salvador | 12(10,15) | 0.28(0.23,0.34) | 22(18,26) | 0.33(0.27,0.40) | -11.10(-15.31,-6.93) | 188(147,236) | 5.13(4.06,6.38) | 432(352,525) | 6.99(5.70,8.49) | 5.80(0.87,10.93) | 114(92,140) | 3.08(2.50,3.81) | 349(269,438) | 5.66(4.36,7.07) | 17.34(0.72,41.39) |
| Republic of Equatorial Guinea | 1(1,1) | 0.23(0.18,0.28) | 3(2,4) | 0.23(0.19,0.28) | 1.70(-3.26,7.83) | 10(8,13) | 3.73(2.94,4.69) | 44(35,57) | 4.37(3.47,5.42) | 7.12(1.23,15.00) | 4(3,6) | 1.48(1.05,2.08) | 21(15,29) | 2.23(1.64,3.00) | 38.35(-38.60,188.15) |
| Republic of Estonia | 17(15,19) | 1.05(0.93,1.18) | 11(10,12) | 0.89(0.79,1.00) | 23.45(15.66,32.09) | 296(255,343) | 16.54(14.17,19.30) | 288(251,331) | 17.58(15.19,20.32) | 40.11(29.71,51.06) | 816(743,891) | 45.63(41.51,49.61) | 318(266,368) | 18.52(15.55,21.47) | 26.56(13.20,41.62) |
| Republic of Fiji | 1(1,2) | 0.18(0.15,0.23) | 2(1,2) | 0.18(0.15,0.22) | -1.78(-6.50,3.74) | 13(10,18) | 2.04(1.57,2.63) | 20(15,26) | 2.12(1.65,2.72) | 7.81(2.10,14.48) | 4(2,5) | 0.58(0.38,0.82) | 6(4,8) | 0.60(0.39,0.85) | 7.96(2.16,14.64) |
| Republic of Finland | 131(118,143) | 2.49(2.27,2.74) | 149(138,163) | 2.99(2.76,3.26) | 1.27(-3.46,6.92) | 4056(3670,4499) | 65.89(59.54,72.95) | 6262(5702,6847) | 82.31(74.97,90.69) | 10.55(4.53,17.16) | 2527(2203,2865) | 40.33(35.11,45.91) | 3548(3074,4066) | 44.08(37.61,50.70) | 43.98(-38.81,222.47) |
| Republic of Ghana | 77(65,89) | 0.66(0.57,0.75) | 339(303,378) | 1.08(0.98,1.19) | -2.57(-6.68,1.40) | 1076(889,1279) | 11.42(9.63,13.37) | 5139(4394,5980) | 20.57(17.97,23.55) | 7.28(2.51,12.24) | 1001(499,1939) | 7.28(4.06,13.09) | 3750(2053,7448) | 11.24(6.83,20.35) | 303.12(207.87,468.63) |
| Republic of Guatemala | 19(15,23) | 0.31(0.25,0.38) | 60(50,73) | 0.40(0.33,0.47) | 47.44(38.15,58.25) | 265(207,334) | 5.23(4.17,6.54) | 1041(850,1281) | 7.83(6.42,9.48) | 63.73(52.76,75.70) | 210(185,242) | 4.08(3.58,4.73) | 847(709,1010) | 6.34(5.32,7.56) | 107.71(84.83,132.30) |
| Republic of Guinea | 18(14,22) | 0.38(0.31,0.45) | 43(36,53) | 0.40(0.33,0.48) | -8.22(-15.26,-0.92) | 262(210,325) | 6.34(5.06,7.80) | 618(502,763) | 7.04(5.77,8.52) | 4.79(-1.62,12.25) | 189(95,382) | 3.80(2.09,7.09) | 798(353,1787) | 6.16(3.08,12.82) | 12.12(-39.50,99.61) |
| Republic of Guinea-Bissau | 3(3,4) | 0.43(0.36,0.52) | 8(7,9) | 0.46(0.39,0.54) | 13.12(7.77,18.79) | 43(34,53) | 6.90(5.56,8.44) | 108(88,131) | 7.80(6.46,9.22) | 22.64(15.47,31.16) | 47(22,99) | 5.16(2.69,10.07) | 157(73,345) | 7.43(3.80,15.01) | 1.34(-31.22,45.68) |
| Republic of Guyana | 2(2,3) | 0.31(0.25,0.38) | 3(2,3) | 0.34(0.28,0.41) | 45.67(34.73,57.74) | 30(23,38) | 5.29(4.16,6.58) | 48(39,59) | 6.45(5.26,7.87) | 57.15(45.88,69.79) | 22(18,26) | 3.88(3.32,4.58) | 45(35,58) | 6.01(4.60,7.64) | 90.40(48.80,145.52) |
| Republic of Haiti | 26(21,31) | 0.50(0.41,0.58) | 68(57,80) | 0.53(0.45,0.62) | 3.00(-1.91,8.32) | 369(297,445) | 8.08(6.61,9.72) | 1075(870,1297) | 9.65(7.89,11.48) | 8.00(2.13,14.28) | 281(175,437) | 6.16(3.78,9.52) | 905(550,1478) | 8.22(4.96,13.46) | 26.38(9.09,56.53) |
| Republic of Honduras | 10(8,13) | 0.30(0.24,0.36) | 35(29,43) | 0.36(0.30,0.43) | -5.93(-9.84,-1.85) | 150(117,189) | 5.18(4.12,6.38) | 583(466,714) | 6.95(5.62,8.38) | 9.76(5.06,14.87) | 61(47,81) | 2.15(1.65,2.81) | 237(171,314) | 2.89(2.12,3.78) | 28.59(6.66,57.84) |
| Republic of Iceland | 10(10,11) | 3.79(3.57,3.99) | 13(12,15) | 3.92(3.43,4.52) | 2.30(-2.14,8.00) | 266(250,283) | 101.08(95.22,107.57) | 485(420,568) | 110.57(95.56,129.91) | 9.49(4.47,15.53) | 129(108,147) | 49.47(41.57,56.45) | 246(206,289) | 54.67(45.54,64.89) | 18.60(4.19,38.27) |
| Republic of India | 2864(2339,3507) | 0.37(0.30,0.45) | 6209(5135,7401) | 0.41(0.34,0.49) | 11.08(3.83,17.63) | 46497(37347,57810) | 6.93(5.59,8.50) | 117013(95945,141319) | 8.25(6.76,9.91) | 20.84(13.19,28.01) | 15261(10952,20924) | 2.29(1.64,3.13) | 41636(31004,54604) | 2.95(2.20,3.84) | 28.92(17.74,44.24) |
| Republic of Indonesia | 267(212,339) | 0.17(0.14,0.21) | 499(402,603) | 0.17(0.14,0.21) | 14.27(6.60,24.23) | 3003(2241,4001) | 1.94(1.48,2.48) | 6470(5000,8302) | 2.11(1.64,2.69) | 19.34(10.31,31.37) | 1082(777,1460) | 0.69(0.50,0.93) | 2761(2038,3604) | 0.92(0.68,1.19) | 8.05(-1.34,18.95) |
| Republic of Iraq | 184(150,221) | 1.12(0.93,1.35) | 638(523,762) | 1.47(1.21,1.75) | -11.14(-15.49,-7.16) | 3362(2727,4070) | 29.83(24.37,35.81) | 14433(11753,17250) | 42.01(34.47,50.04) | -8.96(-13.71,-4.86) | 1116(822,1525) | 9.78(7.17,13.39) | 4769(3415,6362) | 13.58(9.71,17.86) | -8.13(-20.40,4.85) |
| Republic of Italy | 1318(1126,1520) | 2.27(1.94,2.64) | 1391(1189,1601) | 2.83(2.45,3.29) | 46.28(35.71,57.74) | 46113(39355,53934) | 65.31(55.74,76.41) | 74249(63945,87630) | 86.52(74.76,101.32) | 67.73(55.86,83.24) | 21129(17665,25200) | 29.64(24.64,35.39) | 33337(27708,39506) | 36.82(30.05,44.55) | 70.68(38.16,112.05) |
| Republic of Kazakhstan | 391(350,434) | 2.65(2.38,2.94) | 582(531,647) | 2.85(2.61,3.16) | 1.18(-2.90,6.07) | 7206(6418,8070) | 52.20(46.50,58.29) | 11625(10523,12859) | 59.21(53.61,65.47) | 8.38(2.84,15.02) | 3622(2997,4253) | 26.24(21.88,30.93) | 4057(3058,5215) | 21.05(15.91,26.81) | 19.57(-43.31,159.84) |
| Republic of Kenya | 38(30,48) | 0.22(0.18,0.27) | 110(89,136) | 0.24(0.20,0.29) | 17.50(12.59,22.80) | 529(410,686) | 3.98(3.14,4.98) | 1686(1325,2135) | 4.52(3.62,5.61) | 28.08(23.24,33.69) | 194(138,266) | 1.49(1.05,2.02) | 722(539,929) | 1.99(1.50,2.57) | 62.87(7.56,160.12) |
| Republic of Kiribati | 0(0,0) | 0.19(0.16,0.23) | 0(0,0) | 0.19(0.16,0.23) | -1.66(-6.26,2.28) | 1(1,1) | 1.57(1.19,2.02) | 2(1,2) | 1.74(1.33,2.22) | 7.20(2.33,12.27) | 0(0,0) | 0.45(0.29,0.63) | 1(0,1) | 0.50(0.32,0.70) | 7.20(2.33,12.30) |
| Republic of Korea | 195(156,240) | 0.40(0.32,0.49) | 219(179,263) | 0.39(0.32,0.47) | 45.51(36.94,55.46) | 3701(2958,4631) | 8.84(7.17,10.97) | 7097(5812,8594) | 9.35(7.69,11.31) | 58.84(50.26,70.01) | 1641(1304,2111) | 3.93(3.13,5.04) | 2603(1945,3406) | 3.45(2.57,4.51) | 13.02(4.36,21.54) |
| Republic of Latvia | 32(28,35) | 1.16(1.04,1.29) | 19(17,21) | 1.09(0.99,1.20) | 56.55(43.91,70.22) | 525(453,606) | 17.18(14.79,20.08) | 466(412,530) | 19.85(17.31,22.83) | 68.38(53.78,84.40) | 1570(1437,1711) | 51.38(46.89,56.03) | 667(565,772) | 27.12(22.96,31.31) | 85.24(37.43,161.84) |
| Republic of Liberia | 6(5,8) | 0.34(0.27,0.40) | 17(14,20) | 0.35(0.29,0.41) | 12.33(7.08,17.83) | 89(71,110) | 5.55(4.49,6.82) | 249(204,311) | 6.22(5.09,7.51) | 17.57(12.25,23.60) | 85(42,179) | 3.87(2.15,7.60) | 366(154,825) | 6.35(2.90,13.56) | 26.52(14.98,41.12) |
| Republic of Lithuania | 42(38,47) | 1.11(0.99,1.24) | 27(24,29) | 1.04(0.94,1.14) | 36.36(27.70,45.84) | 678(583,799) | 16.59(14.19,19.71) | 659(581,742) | 18.97(16.52,21.68) | 43.38(33.99,52.88) | 1953(1736,2191) | 48.29(42.92,54.09) | 982(826,1137) | 27.22(22.75,31.59) | 66.33(26.59,113.44) |
| Republic of Madagascar | 31(25,39) | 0.32(0.26,0.40) | 79(65,97) | 0.32(0.26,0.38) | 19.26(13.69,25.07) | 443(350,566) | 5.77(4.57,7.24) | 1210(963,1509) | 5.94(4.77,7.23) | 32.53(26.06,39.80) | 162(112,230) | 2.15(1.47,3.03) | 484(327,684) | 2.42(1.61,3.41) | 79.98(48.76,114.41) |
| Republic of Malawi | 22(18,28) | 0.29(0.24,0.36) | 48(39,59) | 0.29(0.24,0.35) | -4.49(-9.35,0.44) | 304(237,385) | 4.91(3.83,6.10) | 675(537,856) | 5.22(4.15,6.44) | 3.04(-1.95,8.46) | 109(75,156) | 1.80(1.22,2.52) | 282(181,412) | 2.25(1.41,3.27) | 3.15(-1.90,8.55) |
| Republic of Maldives | 0(0,0) | 0.15(0.12,0.19) | 1(1,1) | 0.13(0.11,0.16) | 6.14(0.57,12.96) | 3(2,3) | 1.77(1.33,2.29) | 11(9,15) | 1.87(1.43,2.35) | 12.37(6.34,19.33) | 1(1,1) | 0.62(0.44,0.89) | 4(3,6) | 0.73(0.51,0.99) | 42.29(19.97,73.40) |
| Republic of Mali | 29(24,35) | 0.45(0.36,0.53) | 82(69,99) | 0.45(0.38,0.53) | 29.23(22.22,38.27) | 430(349,533) | 7.61(6.17,9.38) | 1170(960,1411) | 8.15(6.76,9.63) | 45.49(37.16,55.44) | 381(168,824) | 5.12(2.53,10.26) | 1654(624,3570) | 7.09(3.25,13.98) | 66.07(34.26,109.24) |
| Republic of Malta | 3(3,4) | 0.84(0.74,0.97) | 4(4,5) | 1.04(0.90,1.19) | -4.31(-6.19,-2.21) | 79(69,93) | 19.00(16.41,22.35) | 162(136,189) | 26.63(22.36,31.29) | 4.03(1.90,6.57) | 57(50,67) | 13.60(11.86,15.69) | 112(97,130) | 17.21(14.74,20.26) | 18.53(-2.24,54.83) |
| Republic of Mauritius | 2(2,3) | 0.20(0.16,0.25) | 3(2,4) | 0.20(0.16,0.24) | 34.10(25.94,43.85) | 27(20,35) | 2.59(2.01,3.30) | 44(35,55) | 2.77(2.17,3.50) | 50.09(41.40,60.50) | 8(5,11) | 0.73(0.48,1.03) | 46(40,52) | 2.95(2.59,3.38) | 58.21(13.65,127.72) |
| Republic of Moldova | 20(16,24) | 0.44(0.35,0.54) | 18(15,21) | 0.47(0.39,0.56) | -12.42(-17.20,-5.89) | 260(206,328) | 5.63(4.44,7.08) | 412(336,507) | 8.80(7.14,10.83) | 7.95(-0.42,18.61) | 458(412,508) | 10.03(9.00,11.09) | 332(278,394) | 6.85(5.71,8.16) | -39.46(-45.10,-33.52) |
| Republic of Mozambique | 34(27,42) | 0.32(0.26,0.40) | 81(67,101) | 0.33(0.28,0.40) | -1.96(-10.67,7.91) | 491(387,619) | 5.45(4.31,6.80) | 1145(923,1429) | 5.89(4.76,7.17) | 2.90(-7.26,13.36) | 175(118,252) | 1.96(1.31,2.84) | 472(297,693) | 2.48(1.55,3.61) | -0.15(-10.56,11.36) |
| Republic of Namibia | 4(3,5) | 0.32(0.26,0.38) | 8(6,9) | 0.32(0.27,0.39) | 20.59(13.16,28.56) | 54(43,68) | 5.77(4.63,7.15) | 127(105,159) | 6.32(5.12,7.79) | 25.07(16.70,33.86) | 18(13,25) | 1.97(1.43,2.69) | 46(33,62) | 2.34(1.71,3.14) | 37.06(5.65,81.36) |
| Republic of Nauru | 0(0,0) | 0.16(0.13,0.20) | 0(0,0) | 0.16(0.12,0.19) | 29.49(22.15,39.98) | 0(0,0) | 1.43(1.08,1.87) | 0(0,0) | 1.56(1.18,1.99) | 40.02(30.84,52.18) | 0(0,0) | 0.41(0.26,0.59) | 0(0,0) | 0.44(0.29,0.64) | 11.25(-6.07,30.94) |
| Republic of Nicaragua | 9(7,11) | 0.32(0.26,0.38) | 31(26,37) | 0.46(0.39,0.55) | 59.30(40.70,79.91) | 132(105,165) | 5.97(4.79,7.29) | 594(494,716) | 10.02(8.35,11.99) | 72.54(51.90,95.29) | 81(65,100) | 3.55(2.86,4.43) | 362(284,459) | 6.05(4.73,7.62) | 71.19(44.18,106.74) |
| Republic of Niue | 0(0,0) | 0.20(0.16,0.25) | 0(0,0) | 0.19(0.16,0.23) | 9.27(3.53,15.25) | 0(0,0) | 2.35(1.81,3.00) | 0(0,0) | 2.46(1.92,3.12) | 13.02(5.54,19.18) | 0(0,0) | 0.66(0.43,0.95) | 0(0,0) | 0.70(0.46,0.98) | 11.76(-49.54,140.80) |
| Republic of Palau | 0(0,0) | 0.16(0.12,0.19) | 0(0,0) | 0.15(0.12,0.19) | 3.58(-0.77,8.43) | 0(0,0) | 1.68(1.27,2.17) | 0(0,1) | 1.77(1.36,2.29) | 10.05(5.05,15.43) | 0(0,0) | 0.47(0.31,0.69) | 0(0,0) | 0.50(0.33,0.72) | 50.39(-39.27,272.52) |
| Republic of Panama | 6(5,7) | 0.27(0.22,0.32) | 14(12,17) | 0.32(0.26,0.38) | -2.94(-7.78,1.54) | 96(77,119) | 5.16(4.17,6.26) | 301(247,364) | 6.84(5.60,8.25) | 5.79(-0.48,11.24) | 70(60,82) | 3.74(3.21,4.36) | 296(238,357) | 6.73(5.39,8.12) | -12.27(-20.42,-4.74) |
| Republic of Paraguay | 20(17,25) | 0.61(0.51,0.73) | 48(40,57) | 0.65(0.54,0.77) | 7.66(-1.08,20.54) | 368(303,450) | 13.42(11.08,16.27) | 1003(831,1205) | 15.08(12.49,18.07) | 56.18(39.63,80.91) | 141(106,184) | 5.14(3.89,6.75) | 482(378,622) | 7.32(5.76,9.38) | -31.66(-41.91,-20.86) |
| Republic of Peru | 59(48,73) | 0.32(0.26,0.39) | 159(129,190) | 0.42(0.34,0.49) | -3.31(-7.32,2.16) | 974(773,1222) | 6.36(5.14,7.94) | 3360(2705,4064) | 9.26(7.50,11.15) | 8.51(4.18,14.79) | 493(389,628) | 3.24(2.53,4.12) | 1942(1486,2511) | 5.38(4.12,6.97) | 8.61(4.42,14.84) |
| Republic of Poland | 990(864,1131) | 2.52(2.21,2.85) | 754(681,827) | 2.21(2.02,2.41) | -4.89(-9.35,-0.57) | 22244(18952,25889) | 53.30(45.44,62.09) | 27978(25456,30627) | 57.54(52.21,62.97) | 4.82(-0.38,10.58) | 25220(23284,27375) | 59.96(55.30,65.09) | 19406(16962,21728) | 36.30(31.28,41.00) | 5.66(0.73,11.31) |
| Republic of Rwanda | 13(10,16) | 0.24(0.19,0.29) | 28(22,35) | 0.23(0.19,0.28) | -2.39(-6.91,1.93) | 175(136,225) | 3.91(3.07,4.88) | 425(329,546) | 4.18(3.31,5.25) | 5.85(0.52,10.69) | 70(52,96) | 1.60(1.16,2.18) | 182(125,252) | 1.84(1.26,2.52) | 6.15(1.00,11.13) |
| Republic of San Marino | 0(0,0) | 1.36(1.11,1.62) | 0(0,0) | 1.42(1.17,1.70) | 4.65(-0.99,10.10) | 11(9,13) | 37.54(30.83,45.29) | 19(16,23) | 41.26(33.96,49.80) | 9.89(3.88,15.63) | 3(2,4) | 9.78(6.37,13.92) | 5(3,7) | 10.72(7.16,15.23) | 9.65(-6.15,30.64) |
| Republic of Senegal | 23(19,28) | 0.40(0.33,0.47) | 59(49,71) | 0.43(0.36,0.50) | 13.78(7.99,21.03) | 326(262,403) | 7.01(5.64,8.63) | 917(753,1108) | 8.08(6.66,9.62) | 20.14(13.18,28.13) | 339(142,778) | 4.94(2.49,10.20) | 1175(510,2763) | 7.05(3.55,15.37) | 83.22(-28.92,345.05) |
| Republic of Serbia | 163(147,178) | 1.65(1.49,1.83) | 166(153,179) | 1.99(1.83,2.14) | -13.04(-16.09,-9.87) | 4037(3569,4551) | 36.57(32.13,41.47) | 5753(5231,6316) | 50.47(45.55,55.84) | 4.79(-0.19,9.55) | 4258(3135,5880) | 37.94(28.04,52.27) | 4685(3489,6048) | 38.42(28.76,49.45) | -42.33(-51.11,-33.37) |
| Republic of Seychelles | 0(0,0) | 0.20(0.17,0.24) | 0(0,0) | 0.23(0.19,0.27) | -12.56(-19.35,-4.27) | 2(1,2) | 2.41(1.88,3.06) | 4(3,5) | 3.06(2.44,3.78) | 19.55(7.27,34.77) | 1(0,1) | 0.80(0.56,1.08) | 2(1,2) | 1.24(0.90,1.67) | -26.60(-33.17,-19.75) |
| Republic of Sierra Leone | 12(9,14) | 0.34(0.28,0.42) | 28(23,34) | 0.36(0.30,0.43) | -3.43(-8.06,0.41) | 164(131,205) | 5.79(4.68,7.09) | 399(320,492) | 6.40(5.20,7.72) | 7.10(1.70,12.52) | 128(63,299) | 3.36(1.86,7.01) | 540(233,1183) | 5.58(2.83,11.28) | 15.30(-5.33,42.38) |
| Republic of Singapore | 7(6,9) | 0.20(0.16,0.24) | 14(11,17) | 0.20(0.16,0.24) | 26.89(18.44,35.39) | 134(104,172) | 4.31(3.42,5.47) | 370(298,464) | 4.55(3.68,5.72) | 38.37(28.03,48.50) | 56(43,72) | 1.82(1.41,2.34) | 129(95,172) | 1.60(1.18,2.11) | -7.00(-23.58,10.78) |
| Republic of Slovenia | 36(33,40) | 1.72(1.57,1.90) | 35(32,39) | 1.84(1.70,2.02) | 13.18(6.45,19.98) | 857(747,978) | 37.25(32.39,42.62) | 1265(1147,1398) | 44.47(39.63,50.04) | 22.48(14.87,30.87) | 1178(1068,1293) | 50.60(45.76,55.59) | 884(740,1054) | 28.54(23.52,33.94) | 19.95(1.69,41.00) |
| Republic of South Africa | 123(100,152) | 0.36(0.30,0.44) | 221(181,265) | 0.36(0.30,0.43) | 15.20(9.49,21.96) | 2034(1627,2518) | 7.23(5.87,8.88) | 4348(3573,5312) | 7.61(6.29,9.16) | 21.83(15.19,29.75) | 1475(1155,1838) | 5.53(4.28,6.82) | 3416(2818,4127) | 6.20(5.13,7.49) | 33.21(14.84,55.94) |
| Republic of South Sudan | 11(9,15) | 0.25(0.20,0.30) | 19(15,24) | 0.25(0.20,0.30) | -4.42(-8.09,-0.67) | 157(121,204) | 4.16(3.25,5.30) | 288(224,366) | 4.39(3.48,5.43) | 5.90(1.06,10.77) | 55(37,79) | 1.48(1.02,2.11) | 113(75,163) | 1.75(1.14,2.53) | 6.00(1.16,11.02) |
| Republic of Sudan | 144(117,175) | 0.82(0.68,0.99) | 482(398,580) | 1.10(0.93,1.32) | 11.39(5.51,17.53) | 2605(2128,3137) | 20.17(16.42,24.32) | 9298(7596,11314) | 29.77(24.68,36.06) | 16.47(9.11,23.54) | 922(655,1252) | 7.08(5.06,9.60) | 4030(3046,5281) | 12.65(9.60,16.25) | 41.33(-19.57,150.06) |
| Republic of Suriname | 1(1,1) | 0.30(0.24,0.36) | 2(2,2) | 0.34(0.28,0.41) | 35.90(26.73,43.94) | 17(14,22) | 5.51(4.41,6.82) | 44(36,53) | 6.85(5.57,8.37) | 48.59(38.96,57.50) | 15(12,19) | 4.77(3.74,5.75) | 42(30,56) | 6.51(4.66,8.71) | 64.01(36.73,99.88) |
| Republic of Tajikistan | 32(27,38) | 0.82(0.68,0.97) | 73(61,85) | 0.77(0.64,0.90) | 8.31(3.09,14.23) | 529(441,626) | 16.12(13.30,18.99) | 1262(1059,1482) | 15.89(13.33,18.62) | 15.26(9.83,22.34) | 190(140,255) | 5.85(4.35,7.82) | 437(314,598) | 5.62(4.08,7.63) | 42.59(-47.00,239.99) |
| Republic of the Congo | 4(4,6) | 0.24(0.19,0.29) | 12(10,15) | 0.24(0.20,0.29) | 19.99(14.21,26.57) | 61(47,79) | 3.86(3.01,4.87) | 190(150,239) | 4.21(3.36,5.20) | 38.00(29.39,48.27) | 26(19,35) | 1.70(1.24,2.21) | 93(68,121) | 2.14(1.57,2.75) | 1.27(-30.71,41.61) |
| Republic of Gambia | 3(2,4) | 0.38(0.31,0.46) | 9(7,11) | 0.43(0.37,0.51) | 12.95(7.42,19.60) | 39(31,49) | 6.61(5.33,8.10) | 128(104,157) | 7.94(6.58,9.59) | 27.00(18.19,36.29) | 38(16,84) | 4.17(2.19,8.19) | 199(75,438) | 7.64(3.41,15.60) | 54.73(30.79,89.65) |
| Republic of the Marshall Islands | 0(0,0) | 0.18(0.14,0.22) | 0(0,0) | 0.18(0.14,0.21) | 4.96(-0.51,9.92) | 1(0,1) | 1.70(1.28,2.23) | 1(1,1) | 1.83(1.40,2.35) | 10.55(4.10,16.24) | 0(0,0) | 0.48(0.31,0.68) | 0(0,0) | 0.52(0.35,0.74) | 66.17(-36.11,286.65) |
| Republic of the Niger | 24(20,30) | 0.42(0.35,0.50) | 75(62,91) | 0.42(0.35,0.50) | 0.59(-2.83,5.01) | 336(270,418) | 7.11(5.71,8.67) | 1049(866,1283) | 7.70(6.34,9.25) | 5.35(1.34,10.17) | 254(104,568) | 3.95(1.99,7.84) | 1004(363,2258) | 4.73(2.28,9.41) | -11.86(-17.98,-6.59) |
| Republic of the Philippines | 101(81,129) | 0.20(0.16,0.24) | 208(169,254) | 0.19(0.16,0.23) | 10.85(5.42,18.62) | 1167(881,1547) | 2.40(1.85,3.06) | 2707(2093,3469) | 2.50(1.95,3.16) | 23.26(16.25,32.23) | 852(645,1075) | 1.70(1.28,2.12) | 2193(1815,2635) | 2.02(1.67,2.42) | -11.00(-36.84,25.24) |
| Republic of the Union of Myanmar | 81(65,102) | 0.24(0.19,0.29) | 125(102,151) | 0.22(0.18,0.27) | 7.04(2.95,11.81) | 821(624,1086) | 2.41(1.87,3.11) | 1516(1182,1962) | 2.64(2.08,3.40) | 19.38(13.46,27.05) | 304(212,428) | 0.88(0.62,1.25) | 648(460,901) | 1.13(0.80,1.57) | -43.60(-51.73,-34.16) |
| Republic of Trinidad and Tobago | 4(3,5) | 0.34(0.28,0.41) | 6(5,7) | 0.39(0.32,0.46) | 2.82(-1.99,7.58) | 67(53,83) | 6.57(5.27,8.12) | 145(118,173) | 8.35(6.79,10.06) | 16.03(9.45,22.82) | 64(57,73) | 6.44(5.73,7.24) | 161(127,203) | 9.32(7.36,11.80) | 38.59(17.71,68.51) |
| Republic of Tunisia | 112(92,135) | 1.37(1.14,1.65) | 246(208,290) | 2.01(1.70,2.38) | 1.82(-2.13,6.71) | 2324(1896,2836) | 37.04(30.54,44.74) | 7937(6620,9481) | 58.12(48.45,69.41) | 10.91(5.50,18.52) | 791(573,1078) | 12.51(9.14,17.12) | 3153(2334,4108) | 23.06(17.12,29.80) | 10.97(5.65,18.66) |
| Republic of Turkey | 986(933,1039) | 1.72(1.62,1.82) | 1480(1392,1572) | 1.67(1.58,1.77) | -1.67(-5.95,2.65) | 21000(19983,22040) | 47.04(44.75,49.36) | 46303(43987,48478) | 48.32(45.94,50.55) | 1.46(-3.49,6.00) | 7944(6050,10435) | 17.59(13.41,22.86) | 18647(14914,22795) | 19.40(15.50,23.67) | 5.73(-6.42,22.13) |
| Republic of Uganda | 28(22,36) | 0.22(0.18,0.27) | 75(59,95) | 0.22(0.18,0.27) | 0.32(-3.73,3.83) | 376(288,484) | 3.72(2.89,4.67) | 1047(804,1354) | 3.98(3.12,4.96) | 5.38(1.56,9.17) | 128(90,184) | 1.29(0.90,1.83) | 431(291,601) | 1.69(1.14,2.36) | 12.09(-3.87,31.86) |
| Republic of Uzbekistan | 184(159,213) | 1.23(1.06,1.40) | 326(277,374) | 0.92(0.78,1.05) | -0.65(-4.99,3.76) | 3188(2753,3675) | 24.17(20.79,27.74) | 6238(5313,7284) | 19.33(16.47,22.49) | 5.50(0.67,10.72) | 1137(856,1465) | 8.60(6.55,11.06) | 2188(1643,2803) | 7.02(5.36,8.91) | 18.29(2.61,44.90) |
| Republic of Vanuatu | 0(0,0) | 0.19(0.15,0.24) | 1(0,1) | 0.19(0.16,0.23) | 38.27(28.45,48.66) | 2(2,3) | 1.91(1.45,2.52) | 5(4,7) | 2.02(1.56,2.60) | 49.95(38.72,62.45) | 1(0,1) | 0.54(0.35,0.77) | 2(1,2) | 0.57(0.37,0.81) | 27.58(13.41,43.28) |
| Republic of Yemen | 87(71,104) | 0.81(0.67,0.98) | 346(285,413) | 1.07(0.90,1.25) | -1.81(-6.03,2.88) | 1503(1213,1815) | 20.04(16.34,24.10) | 6594(5385,7997) | 28.55(23.58,34.20) | 10.43(3.64,17.42) | 503(352,707) | 6.69(4.71,9.41) | 2611(1897,3668) | 11.31(8.28,15.58) | 9.70(-6.59,29.78) |
| Republic of Zambia | 18(15,23) | 0.31(0.25,0.37) | 51(42,64) | 0.31(0.25,0.37) | 34.19(25.30,43.68) | 240(187,305) | 5.05(3.97,6.25) | 727(580,911) | 5.50(4.44,6.73) | 47.61(37.34,58.77) | 93(65,136) | 1.99(1.35,2.85) | 324(229,444) | 2.53(1.80,3.48) | 78.71(40.33,142.38) |
| Republic of Zimbabwe | 24(19,30) | 0.30(0.24,0.37) | 44(36,53) | 0.32(0.26,0.39) | 14.54(8.81,21.15) | 339(268,427) | 5.39(4.34,6.66) | 657(530,819) | 5.77(4.70,7.03) | 24.23(18.27,32.23) | 96(61,136) | 1.52(1.01,2.12) | 185(123,253) | 1.63(1.09,2.23) | 36.45(1.55,81.82) |
| Romania | 208(180,239) | 0.87(0.75,1.01) | 134(115,153) | 0.76(0.65,0.88) | 26.60(21.73,32.16) | 4165(3496,4866) | 16.28(13.59,19.16) | 4087(3477,4729) | 17.06(14.49,19.89) | 28.26(22.92,33.94) | 6920(6237,7615) | 26.69(24.08,29.36) | 4070(3440,4662) | 15.39(12.99,17.74) | 18.51(8.42,27.02) |
| Russian Federation | 1913(1648,2204) | 1.22(1.05,1.39) | 1352(1219,1494) | 1.06(0.98,1.15) | 6.43(-0.77,16.65) | 41444(35272,48633) | 24.28(20.66,28.42) | 52944(48959,57303) | 29.03(26.84,31.40) | 9.79(1.13,22.70) | 45427(41702,49888) | 26.99(24.77,29.69) | 37459(33133,41915) | 19.81(17.46,22.23) | -11.25(-22.13,-1.00) |
| Saint Kitts and Nevis | 0(0,0) | 0.71(0.61,0.82) | 1(1,1) | 0.90(0.80,1.02) | 42.13(34.08,52.86) | 4(3,5) | 13.03(10.84,15.44) | 14(12,16) | 18.02(15.34,21.25) | 48.21(40.84,59.94) | 7(6,8) | 20.85(19.09,22.87) | 15(12,18) | 19.39(16.07,23.23) | 47.40(20.01,78.35) |
| Saint Lucia | 0(0,1) | 0.38(0.31,0.46) | 1(1,1) | 0.43(0.35,0.51) | 42.06(27.78,60.20) | 7(6,9) | 7.47(5.99,9.08) | 21(17,25) | 9.14(7.47,11.01) | 149.88(120.70,199.89) | 8(7,9) | 8.33(7.50,9.35) | 23(19,27) | 9.99(8.24,12.03) | 137.13(112.41,171.67) |
| Saint Vincent and the Grenadines | 0(0,0) | 0.37(0.31,0.45) | 1(0,1) | 0.43(0.36,0.51) | -6.43(-12.43, -0.05) | 6(5,7) | 7.30(5.91,8.93) | 12(10,14) | 8.89(7.35,10.64) | -1.44(-7.25,5.63) | 4(4,5) | 5.44(4.59,6.34) | 10(8,11) | 7.25(5.97,8.61) | -3.88(-22.56,18.16) |
| Slovak Republic | 56(49,65) | 1.04(0.90,1.20) | 60(54,67) | 1.15(1.04,1.30) | -4.37(-7.66, -0.54) | 1253(1068,1463) | 22.22(18.91,26.05) | 1928(1704,2215) | 27.39(23.93,31.52) | 9.29(4.66,13.89) | 1503(1186,1938) | 26.46(20.94,33.94) | 1780(1355,2365) | 23.55(17.94,31.43) | 26.62(12.41,46.63) |
| Socialist Republic of Viet Nam | 113(91,143) | 0.20(0.17,0.24) | 221(180,262) | 0.21(0.17,0.25) | 28.73(21.65,37.44) | 1289(979,1701) | 2.47(1.90,3.17) | 3204(2512,4032) | 2.86(2.25,3.57) | 44.00(35.41,53.94) | 466(320,670) | 0.88(0.62,1.26) | 1345(962,1857) | 1.22(0.87,1.69) | -3.30(-26.51,29.64) |
| Solomon Islands | 0(0,1) | 0.18(0.14,0.21) | 1(1,1) | 0.18(0.15,0.21) | -0.15(-4.59,4.76) | 4(3,6) | 1.95(1.49,2.50) | 12(9,16) | 2.16(1.68,2.75) | 11.39(6.46,17.42) | 1(1,2) | 0.55(0.36,0.79) | 3(2,5) | 0.61(0.40,0.85) | 31.70(16.27,56.91) |
| State of Eritrea | 8(7,10) | 0.32(0.26,0.39) | 19(16,24) | 0.33(0.27,0.39) | 6.02(0.85,11.69) | 115(90,145) | 5.30(4.21,6.51) | 288(231,360) | 5.74(4.62,7.04) | 12.04(5.42,19.25) | 42(30,61) | 2.01(1.39,2.88) | 124(83,176) | 2.57(1.71,3.62) | 34.88(-46.11,193.44) |
| State of Israel | 55(46,65) | 1.12(0.94,1.31) | 117(99,137) | 1.29(1.09,1.50) | -6.32(-9.72, -2.12) | 1384(1144,1638) | 29.80(24.66,35.20) | 3661(3069,4287) | 36.05(30.10,42.24) | 6.55(2.23,10.98) | 703(584,851) | 15.23(12.64,18.34) | 1763(1453,2104) | 17.08(14.01,20.48) | 7.48(3.13,12.27) |
| State of Kuwait | 23(19,28) | 1.14(0.94,1.36) | 111(91,131) | 1.88(1.59,2.21) | -2.55(-6.67,2.29) | 431(350,522) | 31.53(26.09,37.99) | 3118(2592,3683) | 55.87(47.02,65.88) | 6.16(1.47,10.96) | 115(75,159) | 8.18(5.48,11.36) | 901(647,1214) | 15.94(11.50,21.02) | 6.24(1.39,11.03) |
| State of Libya | 45(37,54) | 1.17(0.99,1.39) | 142(122,169) | 1.76(1.51,2.09) | 14.64(7.85,20.21) | 823(665,992) | 30.81(25.19,37.19) | 3698(3078,4434) | 49.31(41.36,58.75) | 27.09(18.74,33.69) | 305(229,402) | 11.27(8.40,14.78) | 2141(1619,2910) | 27.89(21.31,36.83) | 44.65(14.30,80.12) |
| State of Qatar | 8(6,10) | 1.33(1.08,1.60) | 98(83,115) | 2.11(1.88,2.39) | 46.78(36.30,58.81) | 145(114,180) | 37.99(30.76,46.14) | 2156(1907,2423) | 65.54(58.56,73.57) | 56.89(46.21,69.60) | 40(26,58) | 10.17(6.79,14.42) | 585(405,781) | 17.41(12.36,22.74) | 84.35(49.94,135.93) |
| Sultanate of Oman | 21(17,26) | 1.06(0.89,1.27) | 106(86,126) | 1.67(1.43,1.95) | -2.95(-5.44, -0.03) | 362(291,438) | 27.58(22.86,33.16) | 2103(1726,2559) | 46.43(39.38,55.38) | 2.71(0.23,5.58) | 124(90,168) | 9.41(6.86,12.53) | 794(566,1046) | 17.42(12.34,23.12) | 10.30(-10.59,36.46) |
| Swiss Confederation | 246(221,277) | 3.28(2.96,3.70) | 300(268,337) | 3.49(3.15,3.90) | 6.33(0.27,14.80) | 7373(6475,8533) | 86.46(75.76,100.11) | 11707(10383,13284) | 94.93(83.66,107.26) | 11.23(5.41,18.44) | 4890(4255,5635) | 56.00(48.49,64.74) | 6543(5561,7576) | 49.70(41.79,57.75) | -9.58(-32.88,13.94) |
| Syrian Arab Republic | 127(103,152) | 1.15(0.95,1.37) | 212(178,252) | 1.64(1.37,1.93) | -7.83(-11.92, -3.53) | 2305(1870,2794) | 30.68(25.02,37.11) | 6528(5405,7762) | 45.47(37.54,54.16) | 5.34(0.58,11.22) | 760(557,1005) | 9.95(7.27,13.31) | 2125(1570,2827) | 14.67(10.90,19.66) | 5.41(0.73,11.23) |
| Taiwan (Province of China) | 38(33,45) | 0.19(0.16,0.22) | 68(57,78) | 0.27(0.23,0.31) | 0.35(-3.76,5.20) | 487(415,586) | 2.41(2.07,2.85) | 1883(1574,2225) | 6.01(4.94,7.13) | 7.08(2.26,12.46) | 181(138,233) | 0.91(0.70,1.16) | 686(514,869) | 2.16(1.61,2.76) | 30.50(13.75,59.63) |
| Togolese Republic | 10(8,12) | 0.37(0.30,0.44) | 29(24,35) | 0.39(0.33,0.46) | -6.18(-12.10, -0.60) | 135(107,168) | 6.31(5.07,7.72) | 448(365,542) | 7.07(5.81,8.44) | 3.90(-3.25,11.37) | 164(78,332) | 4.82(2.69,8.86) | 556(247,1162) | 6.50(3.20,12.93) | -29.17(-45.95,-11.22) |
| Tokelau | 0(0,0) | 0.17(0.13,0.21) | 0(0,0) | 0.16(0.13,0.19) | 12.48(5.04,21.12) | 0(0,0) | 1.79(1.37,2.31) | 0(0,0) | 1.91(1.47,2.44) | 9.10(0.77,19.85) | 0(0,0) | 0.51(0.34,0.72) | 0(0,0) | 0.54(0.35,0.77) | 6.05(-12.45,31.72) |
| Turkmenistan | 38(34,44) | 1.50(1.34,1.67) | 83(75,92) | 1.59(1.44,1.76) | 35.43(29.97,42.10) | 648(572,732) | 28.97(25.66,32.42) | 1515(1353,1689) | 32.22(28.88,35.84) | 47.30(41.47,54.97) | 370(305,434) | 16.66(13.83,19.50) | 686(486,870) | 15.06(10.67,19.11) | 24.90(19.58,29.22) |
| Tuvalu | 0(0,0) | 0.17(0.14,0.21) | 0(0,0) | 0.16(0.13,0.19) | 1.29(-3.95,6.18) | 0(0,0) | 1.65(1.25,2.15) | 0(0,0) | 1.74(1.32,2.22) | 6.75(1.63,11.46) | 0(0,0) | 0.47(0.30,0.68) | 0(0,0) | 0.49(0.32,0.69) | 21.08(1.04,48.68) |
| Ukraine | 827(715,930) | 1.50(1.31,1.69) | 627(552,696) | 1.41(1.26,1.58) | 2.62(-5.15,12.14) | 15994(13642,18463) | 25.45(21.65,29.56) | 15285(13419,17455) | 26.44(23.07,30.26) | 5.02(-4.44,15.49) | 19889(18085,22043) | 33.54(30.58,37.05) | 13458(10127,17003) | 23.76(17.82,30.12) | 11.35(4.76,18.86) |
| Union of the Comoros | 1(1,1) | 0.28(0.22,0.34) | 2(2,2) | 0.28(0.23,0.34) | 13.51(7.59,19.71) | 15(12,19) | 5.03(3.98,6.22) | 35(29,43) | 5.33(4.33,6.48) | 22.05(15.23,28.97) | 6(4,8) | 1.94(1.28,2.72) | 16(11,22) | 2.42(1.65,3.31) | 10.23(-24.90,55.57) |
| United Arab Emirates | 25(20,30) | 0.99(0.84,1.15) | 148(120,176) | 1.11(0.99,1.26) | 6.53(1.69,12.38) | 367(291,446) | 22.95(19.00,26.93) | 3566(3070,4177) | 25.04(21.86,28.59) | 9.88(4.29,16.81) | 122(83,172) | 7.87(5.60,10.83) | 1133(795,1525) | 8.35(6.21,10.88) | -15.20(-24.44,-4.42) |
| United Kingdom of Great Britain and Northern Ireland | 1999(1768,2247) | 3.41(3.02,3.85) | 2994(2656,3339) | 4.62(4.08,5.17) | -25.16(-30.42, -19.70) | 55843(48777,63446) | 81.53(70.95,93.35) | 107488(93882,122169) | 120.10(104.09,136.60) | -20.03(-25.22,-14.50) | 40365(36405,44860) | 57.08(51.22,63.55) | 67831(59394,76216) | 71.30(61.83,80.79) | -18.41(-29.87,-6.49) |
| United Mexican States | 302(247,367) | 0.42(0.34,0.50) | 841(720,971) | 0.61(0.52,0.71) | -1.81(-7.24,3.60) | 4734(3800,5842) | 8.00(6.51,9.72) | 18017(14993,21337) | 13.10(10.91,15.51) | 6.21(0.26,11.63) | 3771(3345,4297) | 6.28(5.57,7.18) | 17947(15458,20542) | 13.05(11.24,14.93) | 6.32(0.36,11.89) |
| United Republic of Tanzania | 49(39,63) | 0.25(0.20,0.31) | 126(102,156) | 0.25(0.21,0.31) | 31.26(23.39,41.41) | 696(535,892) | 4.33(3.38,5.44) | 1892(1476,2409) | 4.62(3.68,5.79) | 42.51(33.65,53.37) | 262(177,365) | 1.65(1.11,2.31) | 800(551,1116) | 2.00(1.37,2.77) | 68.96(39.74,114.60) |
| United States of America | 9263(7977,10741) | 3.36(2.92,3.88) | 10634(9771,11599) | 3.45(3.17,3.77) | 0.91(-3.64,6.02) | 271976(232506,318639) | 95.22(81.40,111.69) | 419078(386403,453495) | 100.00(92.10,108.28) | 8.88(3.52,15.89) | 121855(101298,144937) | 43.08(35.89,51.11) | 218867(188098,247278) | 47.97(40.77,55.19) | 27.19(0.62,56.10) |
| United States Virgin Islands | 1(0,1) | 0.48(0.40,0.56) | 0(0,1) | 0.54(0.46,0.63) | 7.73(1.63,14.08) | 10(8,12) | 9.71(7.92,11.64) | 14(12,17) | 11.86(9.75,14.08) | 7.08(0.95,13.87) | 9(7,12) | 8.95(6.42,11.54) | 11(8,14) | 9.87(6.81,13.72) | 7.08(0.97,13.89) |
|  | | | | | | | | | | | | | | | |
