# Supplementary material for: Systematic study and 30-year projections of global and multi-regional burden of multiple sclerosis, 1990–2021
Source: Medicine (Baltimore). 2025 Oct 31;104(44):e45089. doi: 10.1097/MD.0000000000045089 (PMC12582701; doi:10.1097/MD.0000000000045089)

**Figure S1** Age-standardized multiple sclerosis deaths per 100 000 population in 2021 for both sexes, by location


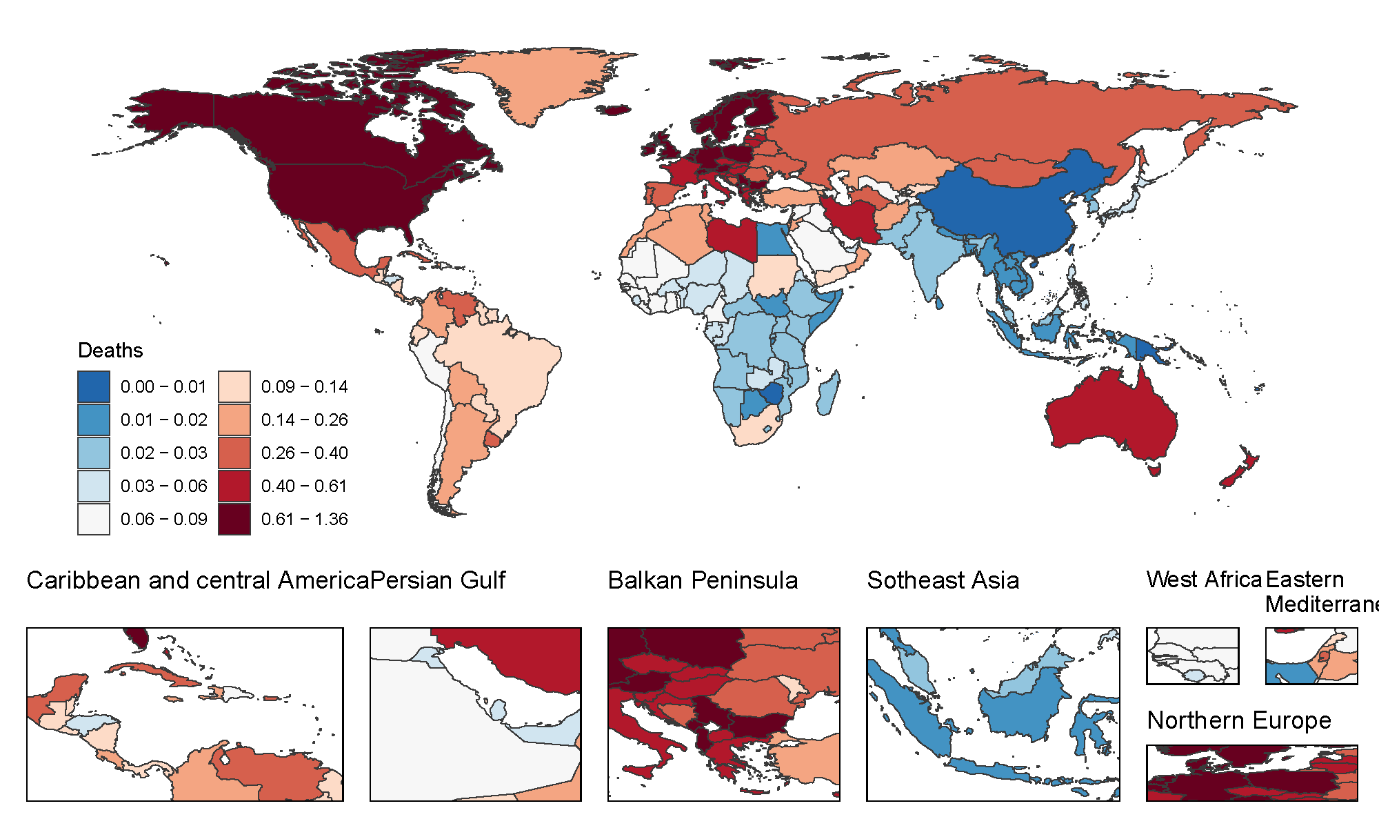


**Figure S2** Age-standardized multiple sclerosis DLAYs per 100 000 population in 2021 for both sexes, by location


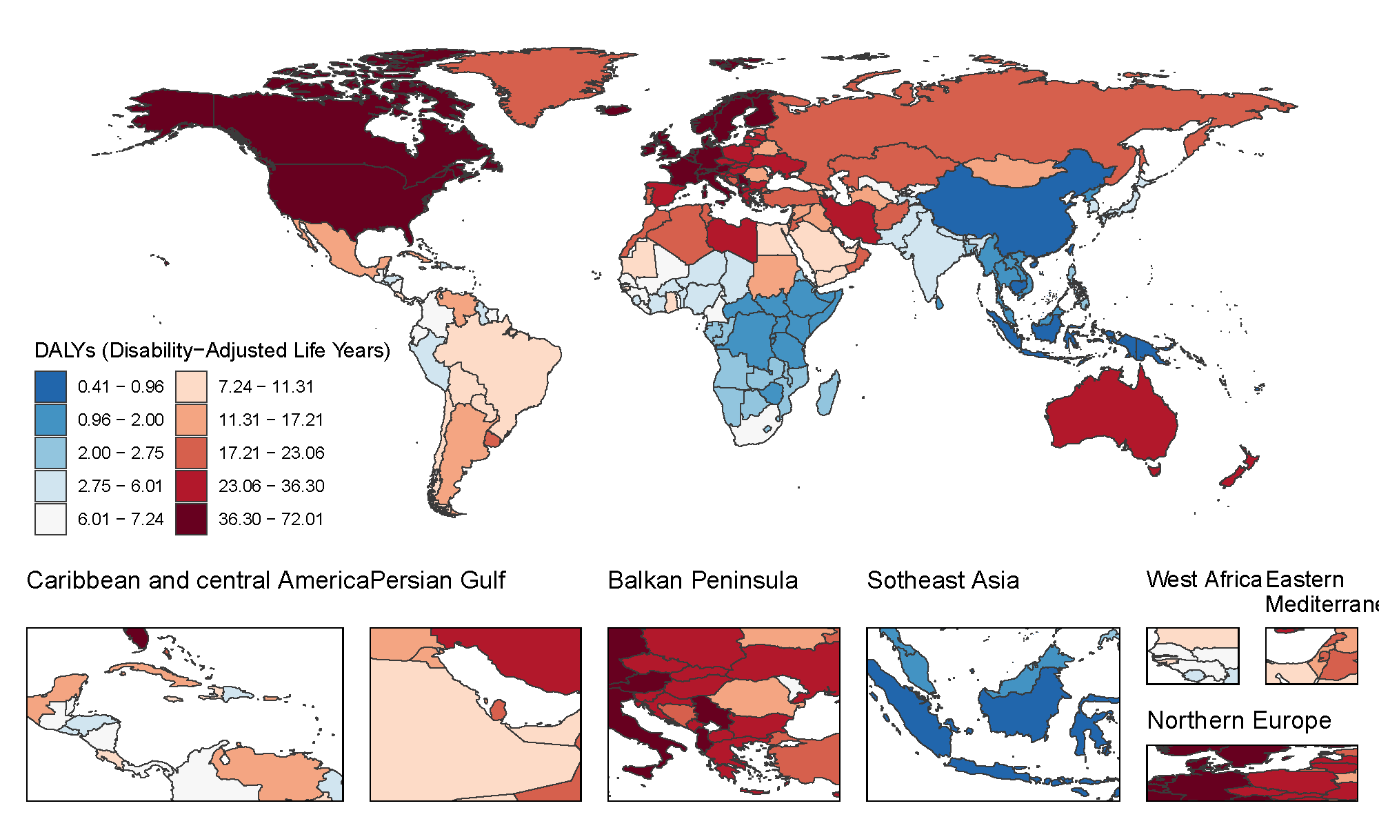


**Figure S3** Age-standardized multiple sclerosis incidence per 100 000 population in 2021 for both sexes, by location


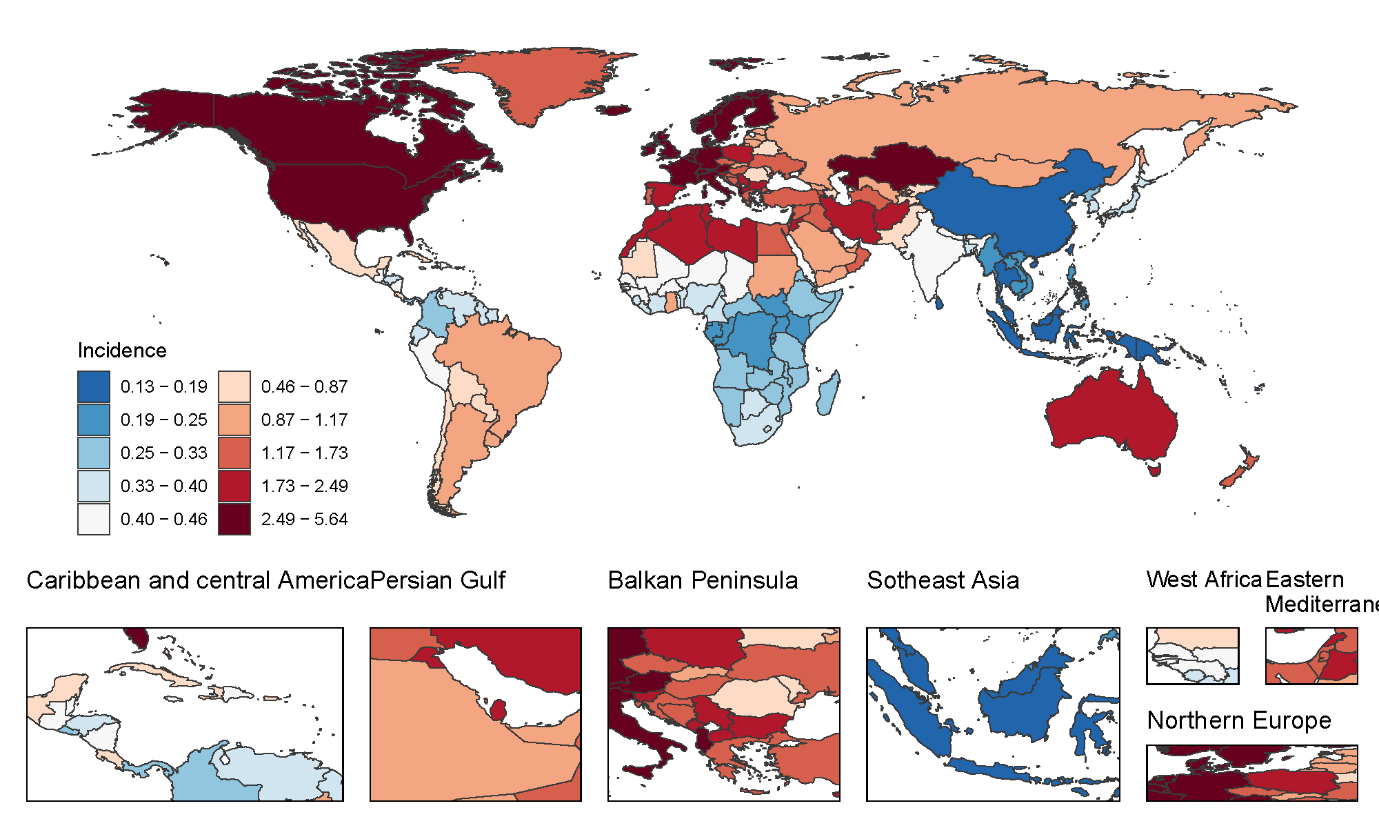


**Figure S4** The prediction of the proportion of each prevalence factor of MS in each age group by 2025 (displayed by age group)


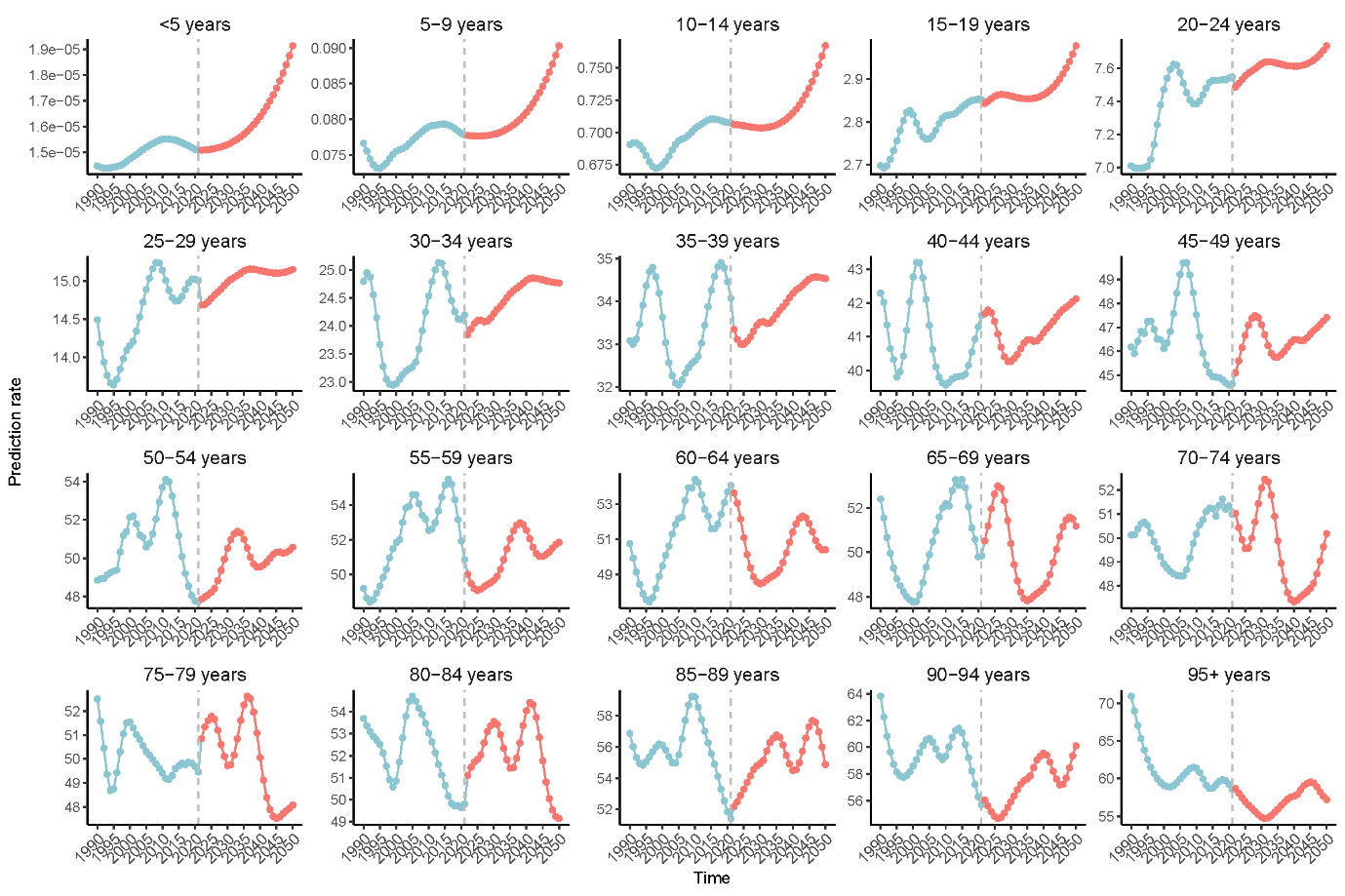


**Figure S5** The prediction of the proportion of each deaths factor of MS in each age group by 2025 (displayed by age group)


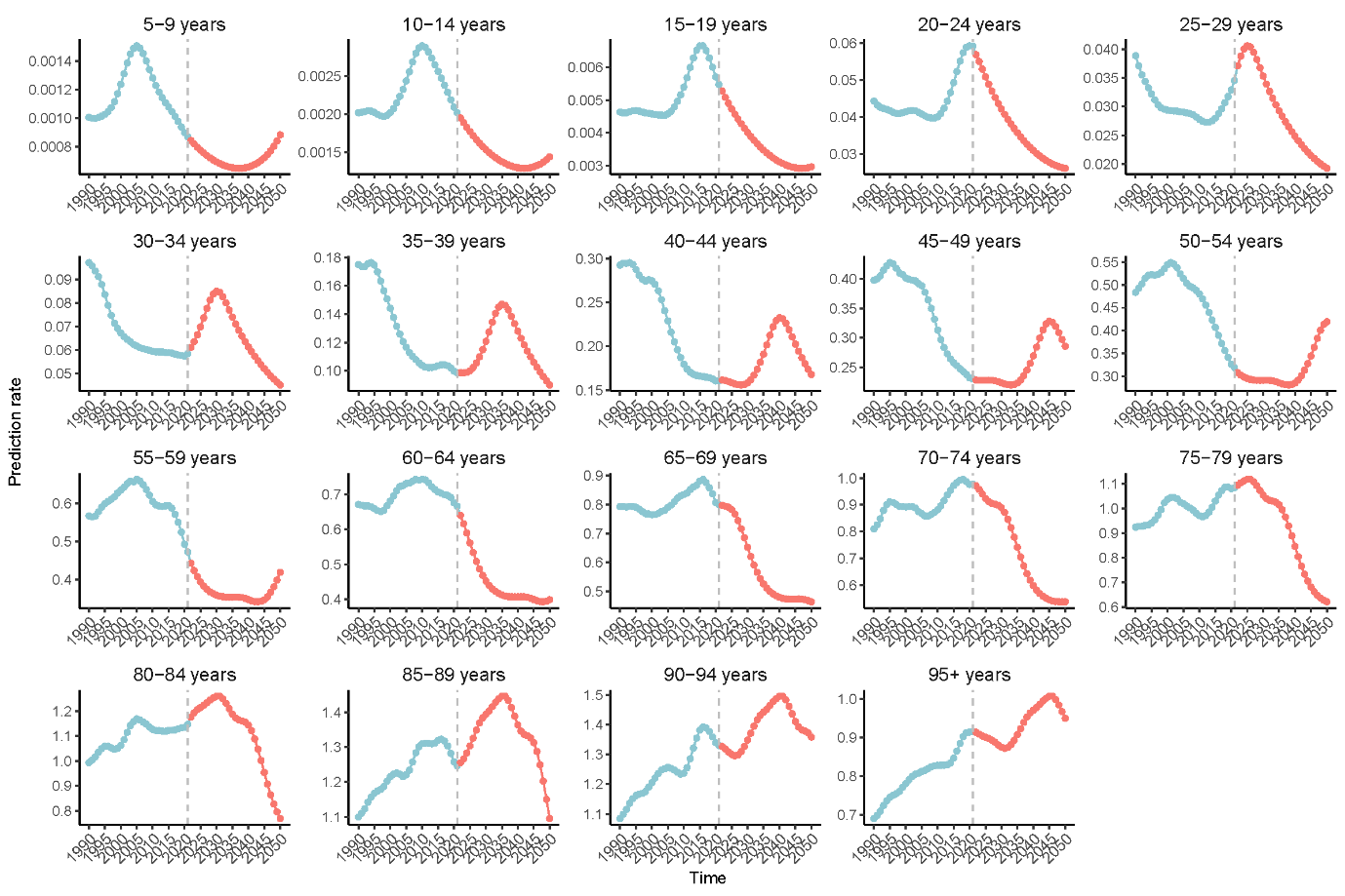


**Figure S6** The prediction of the proportion of each DLAYs factor of MS in each age group by 2025 (displayed by age group)


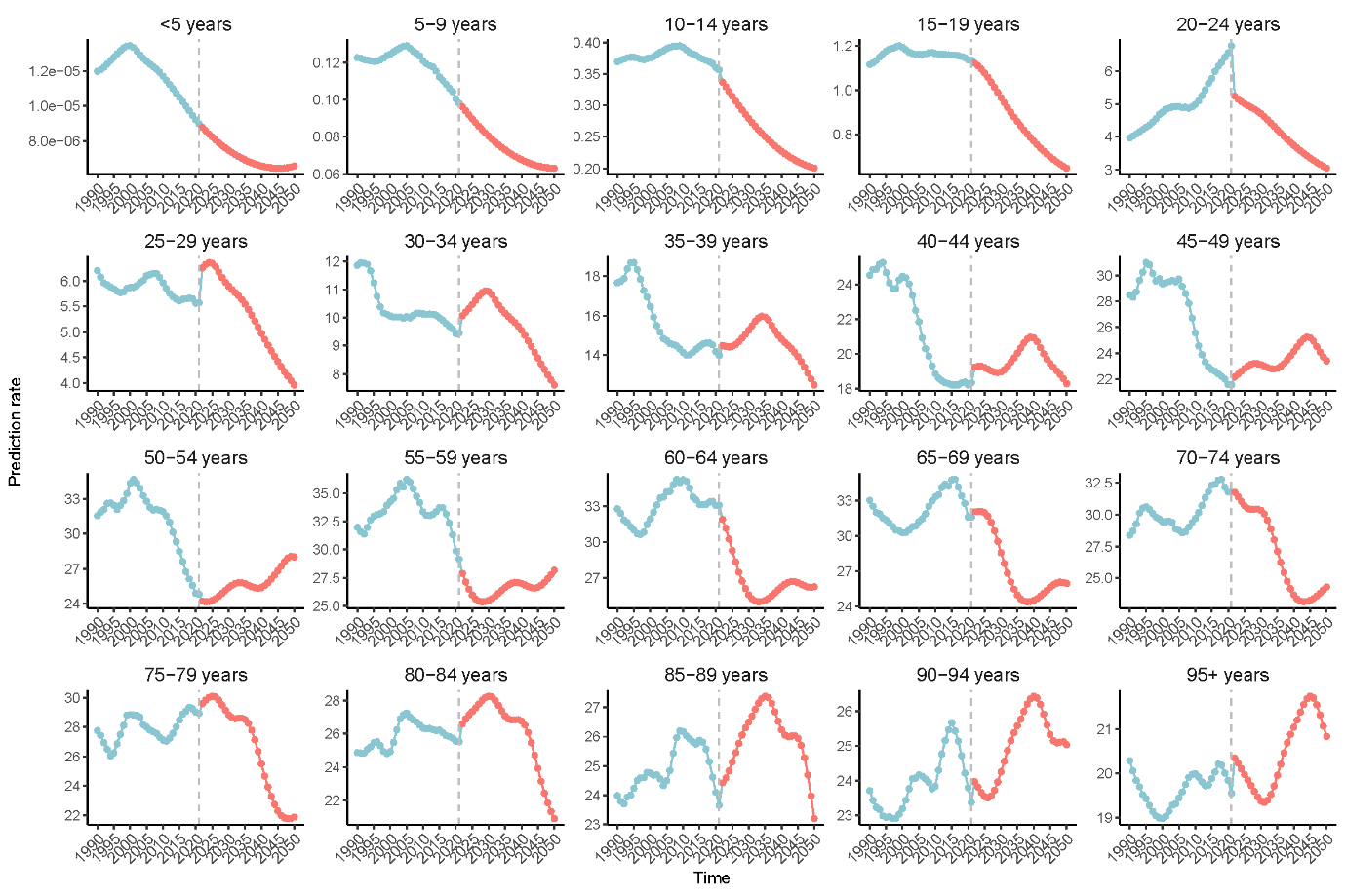


**Figure S7** The prediction of the proportion of each incidence factor of MS in each age group by 2025 (displayed by age group)


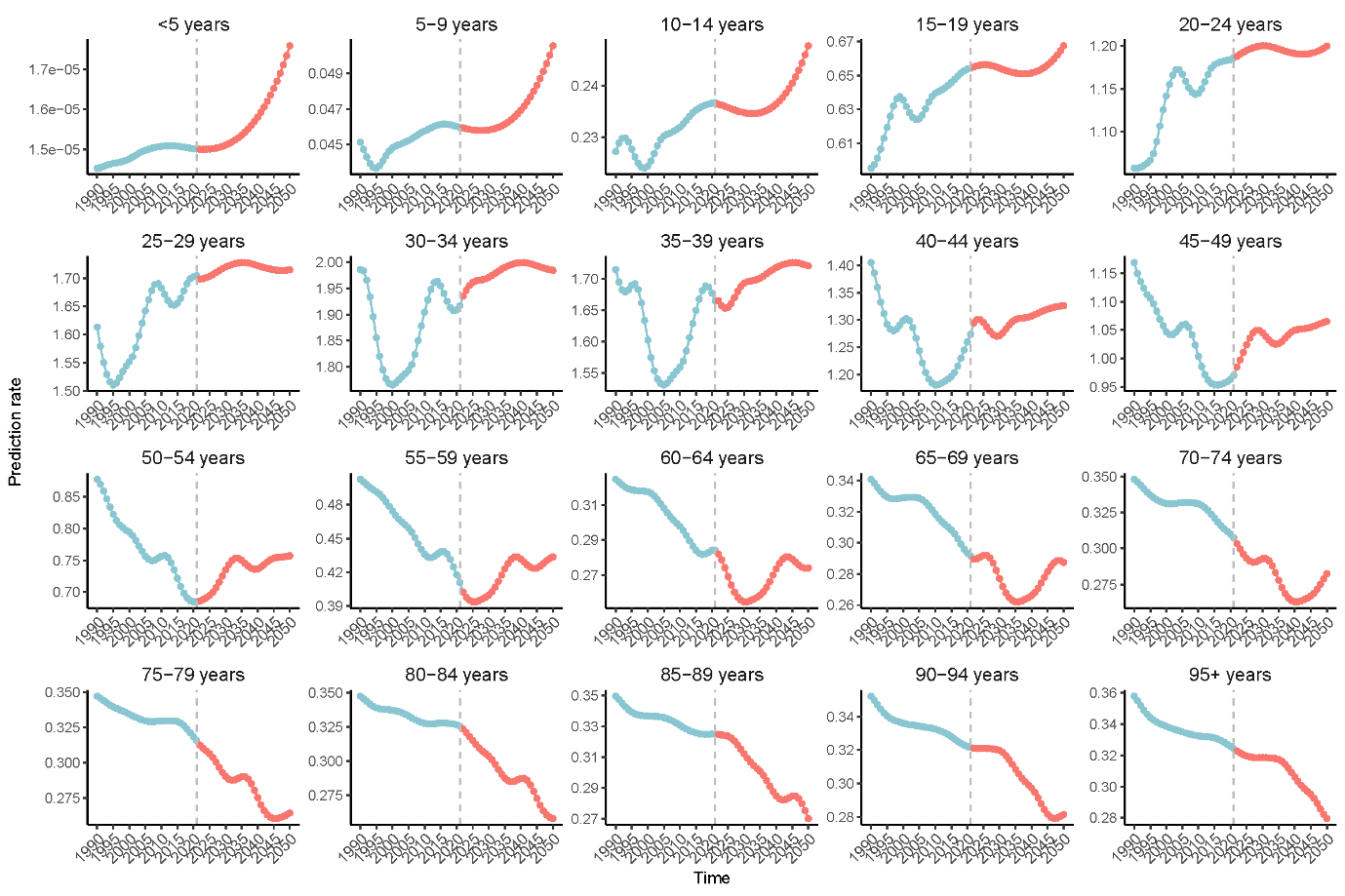


**Figure S8** Frontier analysis of the relationship between different socio-demographic indices (SDI) and deaths. Left panel: Data are shown as lines, with colors representing different years (from 1990 to 2020); Right panel: Data are presented as scatter points, blue scatter points indicate areas with increasing deaths and red scatter points indicate areas with decreasing deaths


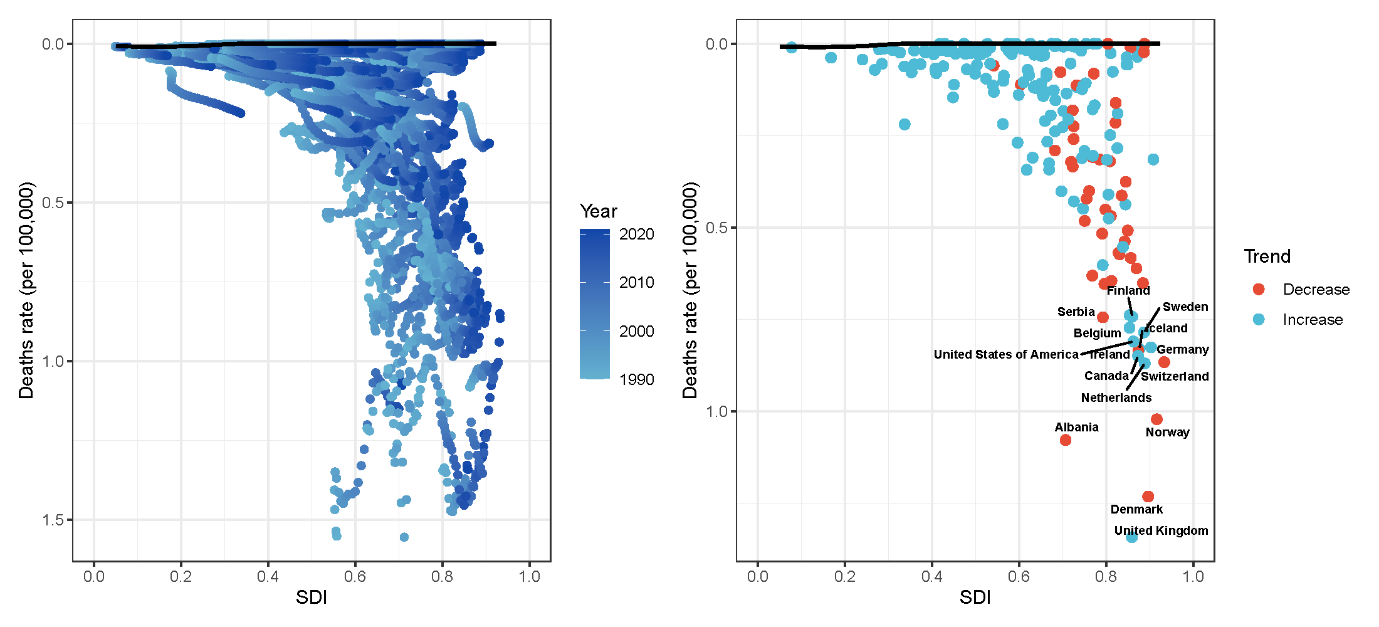


**Figure S9** Frontier analysis of the relationship between different socio-demographic indices (SDI) and DLAYs. Left panel: Data are shown as lines, with colors representing different years (from 1990 to 2020); Right panel: Data are presented as scatter points, blue scatter points indicate areas with increasing DLAYs and red scatter points indicate areas with decreasing DLAYs


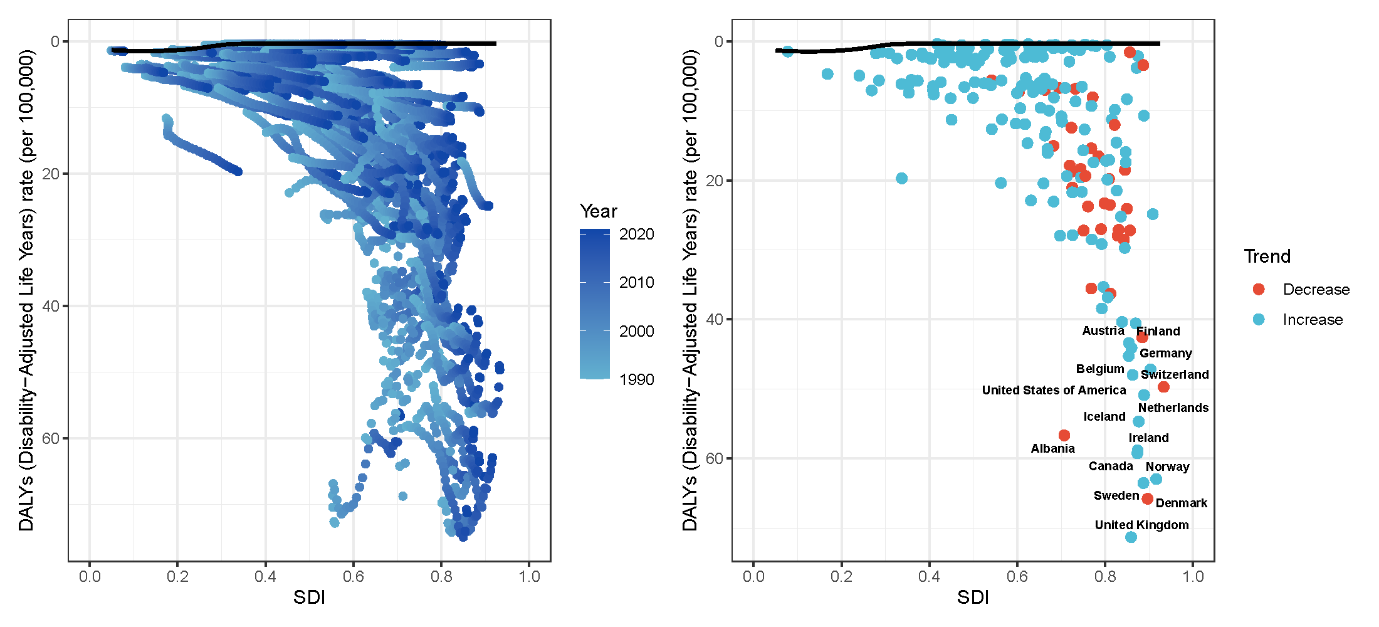


**Figure S10** Frontier analysis of the relationship between different socio-demographic indices (SDI) and incidence. Left panel: Data are shown as lines, with colors representing different years (from 1990 to 2020); Right panel: Data are presented as scatter points, blue scatter points indicate areas with increasing incidence and red scatter points indicate areas with decreasing incidence


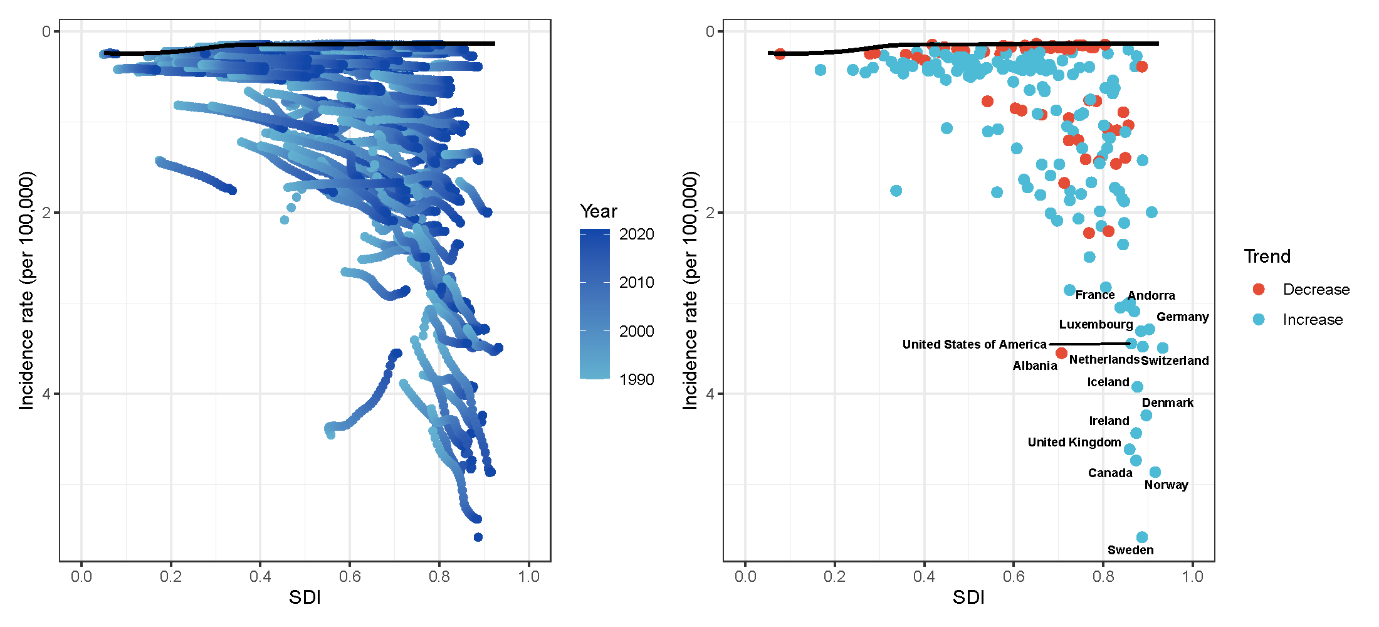

Supplement: Supplementary file 2 [file medi-104-e45089-s002.docx]
